# Supplementary material for: Three-dimensional covalent organic frameworks with pto and mhq-z topologies based on Tri- and tetratopic linkers
Source: Nat Commun. 2023 May 19;14:2865. doi: 10.1038/s41467-023-38538-x (PMC10199027; doi:10.1038/s41467-023-38538-x)
Supplement: Supplementary file 1 — Supplementary Information [file 41467_2023_38538_MOESM1_ESM.pdf]

Supplementary Information

Three-Dimensional Covalent Organic Frameworks with pto and mhq-z Topologies Based on Tri- and Tetratopic Linkers

Dongyang Zhu,[a§] Yifan Zhu,[b§] Yu Chen,[a] Qianqian Yan,[b] Han Wu,[c] Chun-Yen Liu,[a] Xu Wang,[d] Lawrence B. Alemany,[d,e] Guanhui Gao,[b,d] Thomas P Senftle,[a] Yongwu Peng, [f] Xiaowei Wu,[* g] and Rafael Verduzco[* a,b]

[a]Department of Chemical and Biomolecular Engineering, Rice University, 6100 Main Street, MS-362, Houston, Texas 77005, United States

[b]Department of Materials Science and Nanoengineering, Rice University, 6100 Main Street, MS-325, Houston, Texas 77005, United States

[c]Ganjiang Chinese Medicine Innovation Center, Nanchang 330000, China

[d]Shared Equipment Authority, Rice University, 6100 Main Street, Houston, Texas 77005, United States

[e]Department of Chemistry, Rice University, 6100 Main Street, Houston, Texas 77005, United States

[f]College of Materials Science and Engineering, Zhejiang University of Technology, Hangzhou 310014, Zhejiang, China

[g]Xiamen Key Laboratory of Rare Earth Photoelectric Functional Materials, Xiamen Institute of Rare Earth Materials, Fujian Institute of Research on the Structure of Matter, Haixi Institutes, Chinese Academy of Sciences, Xiamen 361021, China

*Corresponding authors. E-mail: xmwuxiaowei@fjirsm.ac.cn; rafaelv@rice.edu

§These authors contribute equally to this work.

Materials

Chemicals. All chemicals were purchased from commercial sources and used without further purification. 1,3,5-Tris(4-aminophenyl)benzene (TAPB), 4',4''',4''''',4'''''''-(ethene-1,1,2,2-tetrayl)tetrakis(([1,1'-biphenyl]-4-carbaldehyde)) (ETTBC), 1,3,6,8-tetrakis(4-aminophenyl)pyrene (Py), and 4',4''',4''''',4'''''''-(ethene-1,1,2,2-tetrayl)tetrakis(([1,1'-biphenyl]-4-amine)) (ETTBA) were purchased from ChemScene. LLC. 1,3,5-Tris(4-formylphenyl)benzene (TFPB) and 5'-(4-aminophenyl)-2',4',6'-trimethyl-[1,1':3',1''-terphenyl]-4,4''-diamine (ATTA) were purchased from Jilin Chinese Academy of Sciences - Yanshen Technology Co., Ltd. 5'-(4-Formylphenyl)-2',4',6'-trimethyl-[1,1':3',1''-terphenyl]-4,4''-dicarbaldehyde (FTTD) was purchased from BLDpharm. Solvents including anhydrous 1-butanol, 1,4-dichlorobenzene (*o*-DCB), dioxane and mesitylene were purchased from Sigma Aldrich. All other solvents used in this work were purchased from VWR.

Instrumentations and methods

Powder X-ray diffraction (PXRD): PXRD data were detected on a Rigaku SmartLab XRD with 2θ ranging from 1° to 30° with 0.02° increment in a continuous mode. Powder samples without prior grinding were directly placed on zero background sample holders and leveled flat using a glass microscope slide.

Fourier-transform infrared spectroscopy (FTIR): FTIR of all solid samples was tested using a Thermo Nicolet iS10 FT-IR spectrometer with a diamond ATR attachment. The spectra were tested using 64 scans with a resolution of 4. The testing range was set from 4000 to 500 cm^{-1}. The spectra are uncorrected.

Nitrogen sorption measurements: Nitrogen sorption measurements were conducted on Quantachrome Autosorb-iQ-MP/Kr BET Surface Analyzer. All samples were degassed at 100 °C for 12 hours and tested directly without grinding. Liquid nitrogen was used to keep the tubes at 77 K. BET surface areas were calculated using BET adsorption models included in the instrument software (ASiQwin version 5.2). Pore size distributions were calculated using the DFT model included in the instrument software.

CO_2 sorption tests: CO_2 sorption tests were also measured using Quantachrome Autosorb-iQ-MP/Kr BET Surface Analyzer. All samples were tested directly without grinding. A chiller was used to keep the BET tubes at 273 K and 298 K.

Scanning electron microscopy (SEM): SEM was performed on an FEI Quanta 400 FESEM operating at 30.00 kV. Samples were prepared by dispersing COF powders in ethanol and dropping them on clean aluminum sample holders. After drying for 24 hours, samples were coated with a 10 nm gold film using a Denton Desk V Sputter.

Transmission electron microscopy (TEM): TEM was performed on a Titan Themis Scan/transmission electron microscope operated at 300 kV. Powder samples were briefly sonicated in ethanol before being dropped on the grids.

Thermogravimetric analysis (TGA): TGA measurements were performed on Q-600 Simultaneous TGA/DSC from TA Instruments under argon atmosphere by heating to 800 °C at a rate of 10 °C min^{-1}.

Solution nuclear magnetic resonance (NMR): Solid state ^{13}C cross-polarization magic angle spinning NMR spectra were obtained on a Bruker 500 MHz spectrometer operated at room temperature.

Elemental analysis: Elemental analysis was conducted on a Costech ECS 4010 Elemental analysis system.

Supercritical CO_2 ($ScCO_2$) drying: Supercritical CO_2 drying was conducted on a Leica EM CPD300 automated Critical Point Dryer. COF powders were thoroughly washed using THF and large access of ethanol. Wetted COF powders were loaded in tea bags and then transferred into the dryer chambers with the addition of an appropriate amount of pure ethanol.

Solid state NMR: Solid state NMR experiments were performed on a Bruker Avance III 200MHz spectrometer using a double-resonance magic-angle spinning (MAS) probe head for 4-mm rotors. The ^{13}C cross-polarization (CP) spectra for COFs RICE-3/4/5/6/7 were obtained with 7.6 kHz MAS, 2.5 ms contact time, and a 5 s relaxation delay. To distinguish between protonated and non-pronated carbon atoms, ^{13}C CP spectra with non-quaternary suppression (CPNQS) with 50 μs and 80 μs dephasing delays were also acquired. For each 13C CP /CPNQS experiment, five to six thousand scans were accumulated. All the spectra were processed with 50 Hz line broadening. Chemical shifts are relative to glycine carbonyl defined as 176.46 ppm.

Crystal structure modeling: Structural modeling of COFs was generated using the Materials Studio program (ver2017) employing the *Visualizer* module, various nets are referenced from the RCSR database (Reticular Chemistry Structure Resource, http://www.rcsr.net). The lattice model was geometrically optimized using a force-field-based method (*Forcite*, UFF, Ewald summations, Qeq). LeBail/Rietveld refinements were performed in EXPO2014. CIFs for RICE-3/7 have been deposited to CCDC database with assigned deposition numbers (2172155; 2172162).

DFT simulation. Periodic DFT simulations were performed using the Vienna *ab initio* Software Package (VASP 5.4.4.).[1,2] The Perdew–Burke–Ernzerhof (PBE) exchange-correlation functional was used.[3] VASP default potentials[4] in the projector augmented wave (PAW)[5] formalism were used to treat the nuclei and frozen-core electrons. Explicit and self-consistent calculations were

performed to treat the following valence electrons: N-$2s^2 2p^3$, O-$2s^2 2p^4$, H-$1s^1$, and C-$2s^2 2p^2$. The kinetic energy cutoff was set at 450 eV to truncate the plane-wave basis sets. Grimme's D3 dispersion[6] was employed to describe the van der Waals interactions. We also tested the dispersion correction term using DFT-D2 approach of Grimme[7,8] and DFT-D3 method with Becke-Johnson damping function[6,9] and found the different methods generate a difference within ±0.2 eV, which justifies our choice of DFT-D3 for dealing with van der Waals interaction. All calculations were spin-polarized. The smearing width of the Gaussian smearing scheme was 0.05 eV. A Monkhorst-Pack (MP)[10] k-point mesh of 1×1×1 was sampled on the unit cell. The bulk structure of COF RICE-7 was first obtained from powder X-ray diffraction (PXRD) measurements. DFT was then applied to optimize the structure with a force convergence criterion of 0.02 eV $Å^{-1}$ and a self-consistent-field electronic energy convergence criteria of 10^{-5} eV. The optimum lattice constants were (32.57 Å, 32.57 Å, 32.57 Å) and (60°, 60°, 60°), determined from cell relaxations in which the volume of the unit cell was allowed to change. The adsorption energy for each structure was estimated by taking the total DFT energy of the adsorption configuration and subtracting the total energy of the reference structure (i.e., the structure with the CO_2 molecule in the center of the largest pore shown in Supplementary Fig. 81).

Synthetic Procedures

1. Synthesis of COF RICE-3

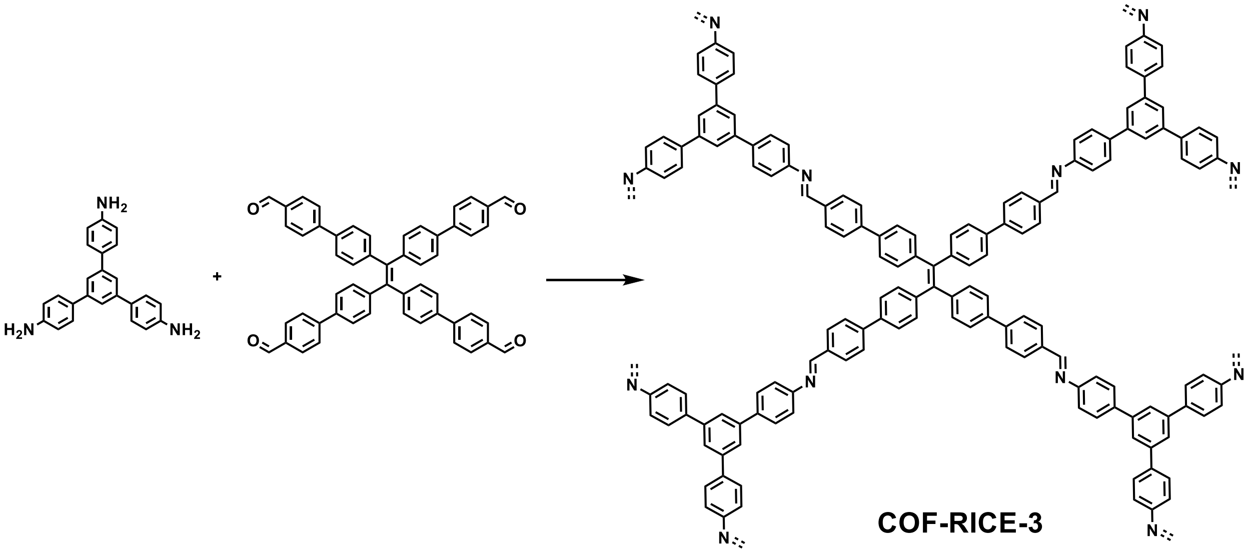

Supplementary Fig. 1. Reaction scheme for the preparation of RICE-3

RICE-3: 1,3,5-Tris(4-aminophenyl)benzene (TAPB) (14.06 mg, 0.04 mmol) and 4',4''',4''''',4'''''''-(ethene-1,1,2,2-tetrayl)tetrakis(([1,1'-biphenyl]-4-carbaldehyde)) (ETTBC) (22.46 mg, 0.03 mmol) were weighed and dissolved in a mixture of 1.6 mL dioxane and 0.4 mL mesitylene in a Pyrex tube directly without degassing. Afterwards, 0.2 mL 6M acetic acid was added and the solution was sonicated for 10 minutes. The tube was further sealed, placed in oven and heated under 120 °C for 3 days. All of the products were separated and washed thoroughly using THF and ethanol. The wet powder samples were sealed in a tea bag and dried using the Leica EM CPD300 Critical Point Dryer.

We also tried to use a solvent mixture of o-dichlorobenzene (o-DCB)/1-butanol (1-BuOH)/ 6M acetic acid (v/v/v, 5/5/1) at the same reaction conditions (120°C, 7 days), however, we could not produce highly crystalline COFs.

2. Synthesis of COF RICE-4/5/6

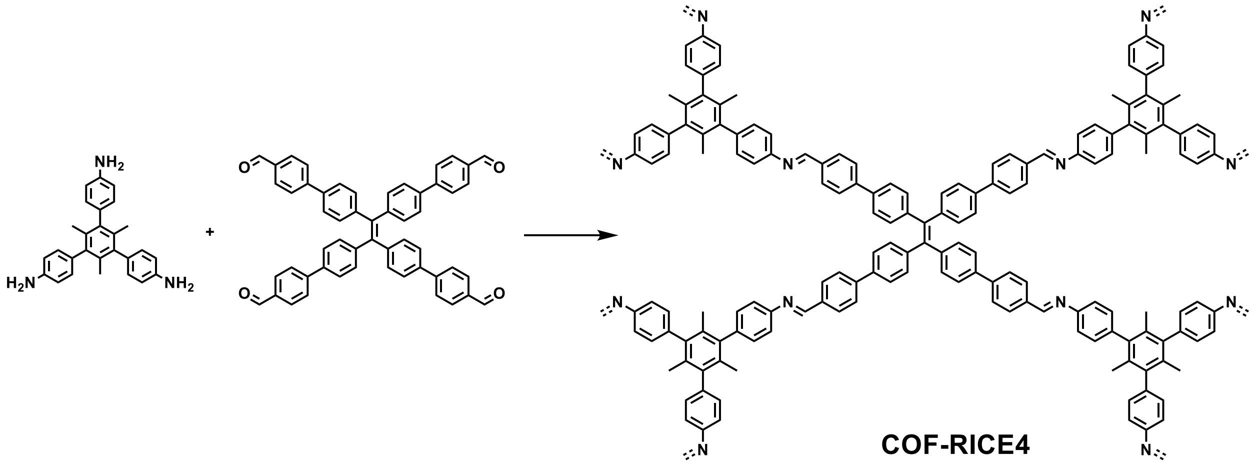

Supplementary Fig. 2. Reaction scheme for the preparation of RICE-4

RICE-4: 5'-(4-Aminophenyl)-2',4',6'-trimethyl-[1,1':3',1''-terphenyl]-4,4''-diamine (ATTA) (7.87 mg, 0.02 mmol) and 4',4''',4''''',4'''''''-(ethene-1,1,2,2-tetrayl)tetrakis(([1,1'-biphenyl]-4-carbaldehyde)) (ETTBC) (11.23 mg, 0.015 mmol) were weighed and dissolved in a mixture of 0.5 mL o-dichlorobenzene (o-DCB) and 0.5 mL 1-butanol (1-BuOH) in a Pyrex tube directly without degassing. Afterwards, 0.2 mL 6M acetic acid was added and the solution was sonicated for 10 minutes. The tube was further sealed, placed in oven and heated under 120 °C for 7 days. All the products were separated and washed thoroughly using THF and ethanol. The wet powder samples were sealed in a tea bag and dried using the Leica EM CPD300 Critical Point Dryer.

We also tried to use a solvent mixture of dioxane/mesitylene/ 6M acetic acid (v/v/v, 4/1/1) under the same reaction conditions (120°C, 7 days), however, the yield of COF powders was very low (less than 3%).

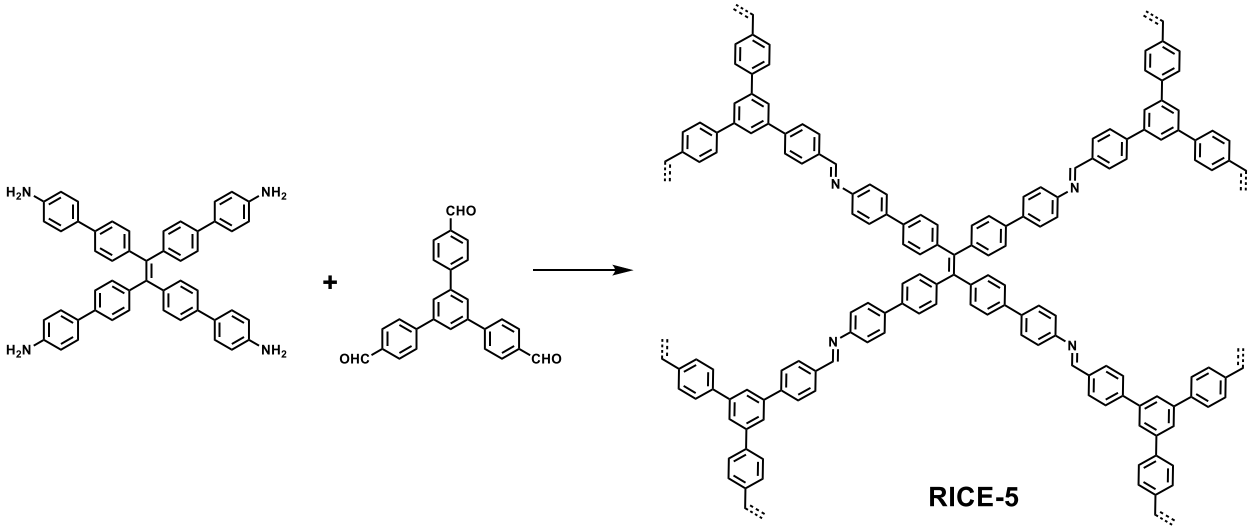

Supplementary Fig. 3. Reaction scheme for the preparation of RICE-5

RICE-5: 4',4''',4''''',4'''''''-(Ethene-1,1,2,2-tetrayl)tetrakis(([1,1'-biphenyl]-4-amine)) (ETTBA) (10.45 mg, 0.015 mmol) and 1,3,5-tris(4-formylphenyl)benzene (TFPB) (7.81 mg, 0.02 mmol) were weighed and dissolved in a mixture of 0.5 mL 0-dichlorobenzene (o-DCB) and 0.5 mL 1-butanol (1-BuOH) in a Pyrex tube directly without degassing. Afterwards, 0.2 mL 6M acetic acid was added and the solution was sonicated for 10 minutes. The tube was further sealed, placed in oven and heated under 120 °C for 7 days. All the products were separated and washed thoroughly using THF and ethanol. The wet powder samples were sealed in a tea bag and dried using the Leica EM CPD300 Critical Point Dryer.

We also tried to use solvent mixture of dioxane/mesitylene/ 6M acetic acid (v/v/v, 4/1/1) under the same reaction conditions (120°C, 7 days), however, this condition could only produce amorphous powders.

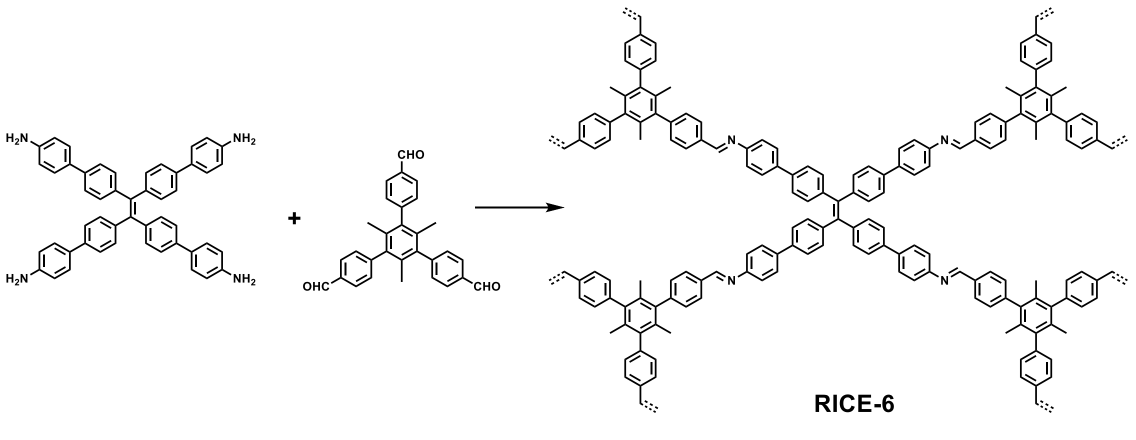

Supplementary Fig. 4. Reaction scheme for the preparation of RICE-6

RICE-6: 4',4''',4''''',4'''''''-(Ethene-1,1,2,2-tetrayl)tetrakis(([1,1'-biphenyl]-4-amine)) (ETTBA) (10.45 mg, 0.015 mmol) and 5'-(4-formylphenyl)-2',4',6'-trimethyl-[1,1':3',1''-terphenyl]-4,4''-dicarbaldehyde (FTTD) (8.65 mg, 0.02 mmol) were weighed and dissolved in a mixture of 0.5 mL *o*-dichlorobenzene (*o*-DCB) and

0.5 mL 1-butanol (1-BuOH) in a Pyrex tube directly without degassing. Afterwards, 0.2 mL 6M acetic acid was added and the solution was sonicated for 10 minutes. The tube was further sealed, placed in oven and heated under 120 °C for 7 days. All of the products were separated and washed thoroughly using THF and ethanol. The wet powder samples were sealed in a tea bag and dried using the Leica EM CPD300 Critical Point Dryer.

We also tried to use solvent mixture of dioxane/mesitylene/ 6M acetic acid (v/v/v, 4/1/1) at the same reaction conditions (120°C, 7 days), however, this condition could only produce amorphous powders.

3. Synthesis of COF RICE-7

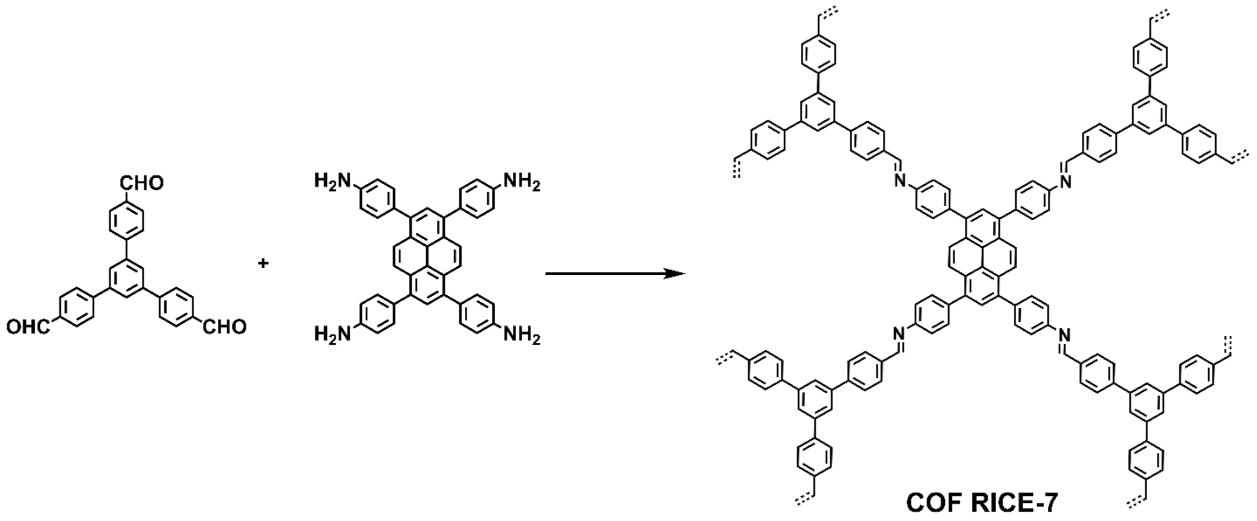

Supplementary Fig. 5. Reaction scheme for the preparation of RICE-7

RICE-7: 1,3,6,8-tetrakis(4-aminophenyl)pyrene (Py) (17.0 mg, 0.03 mmol) and 1,3,5-tri(4-formylphenyl)benzene (TFPB)) (15.6 mg, 0.04 mmol) were weighed and reacted in a mixture of dimethylacetamide/mesitylene/6M acetic acid (5:5:2) in a Pyrex tube. 4 eq. of p-toluidine was added as a modulator which was the key to ensure high crystallinity for the final sample. The tube was further sealed, placed in the oven, and heated under 120 °C for 7 days. The products were separated and washed thoroughly using THF and ethanol. We also tried other synthetic conditions as shown in Supplementary Fig. 14 and the PXRD patterns were also shown there.

Additional Characterization Data

FTIR

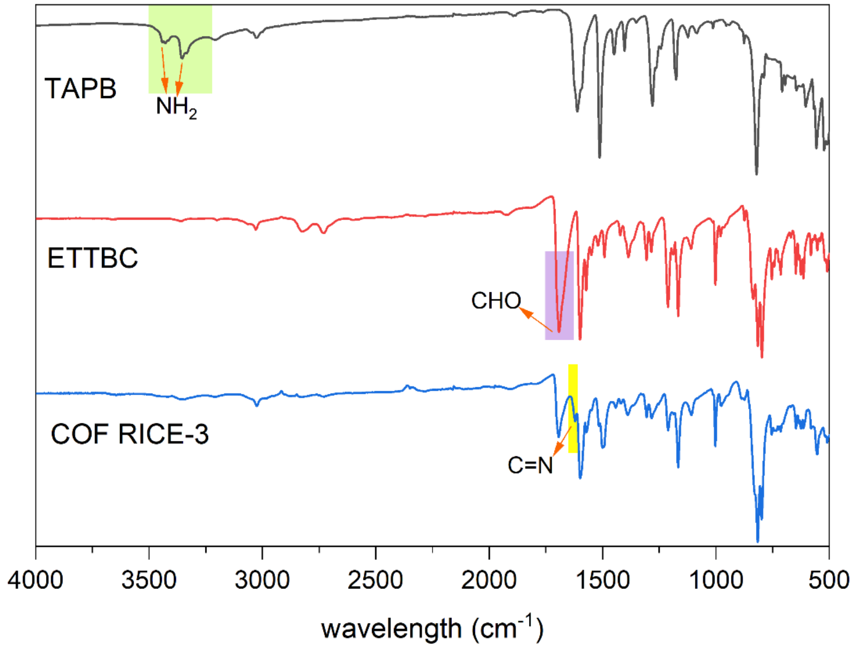

Supplementary Fig. 6. FTIR spectra of COF RICE-3 and its starting monomers.

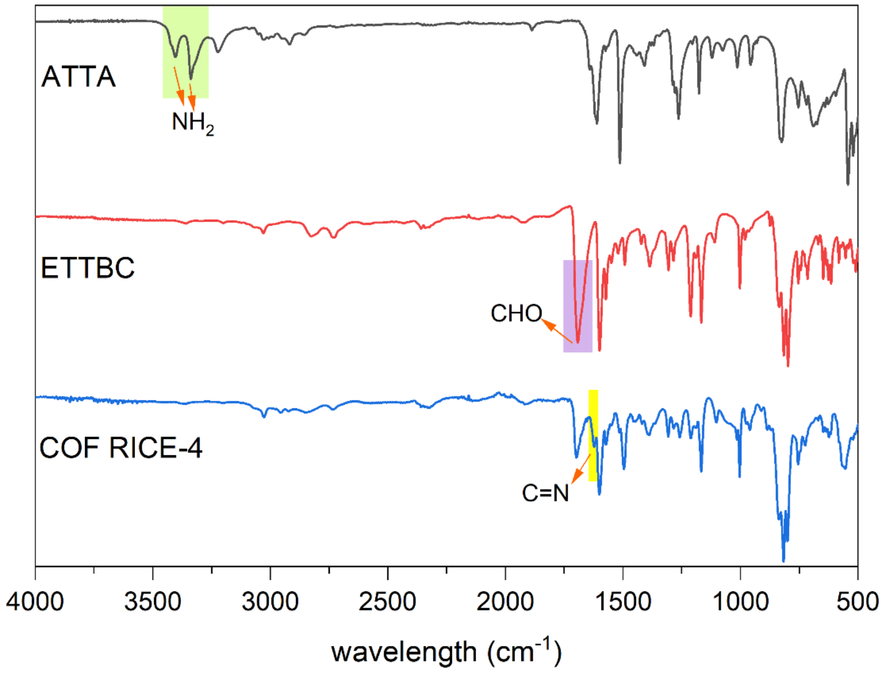

Supplementary Fig. 7. FTIR spectra of COF RICE-4 and its starting monomers.

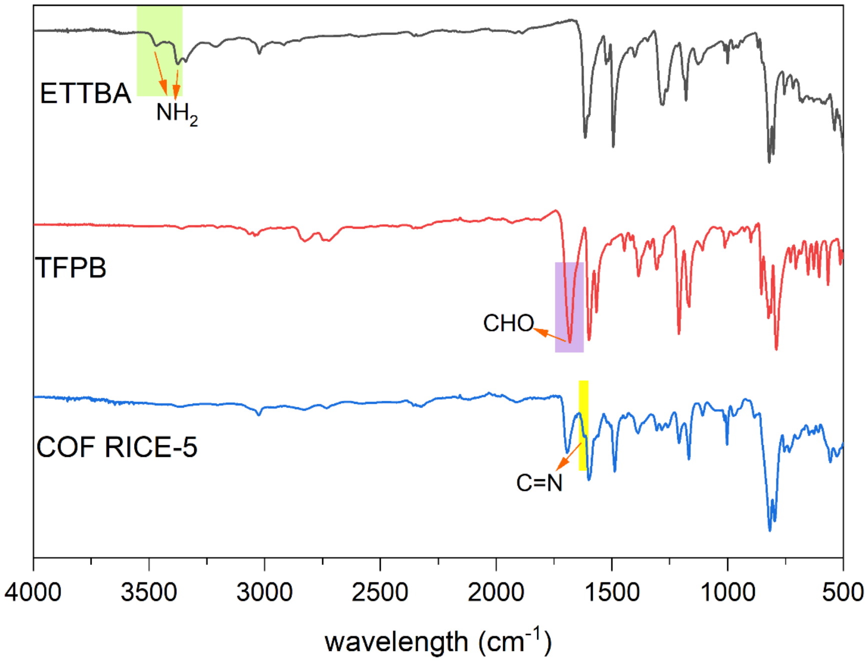

Supplementary Fig. 8. FTIR spectra of COF RICE-5 and its starting monomers.

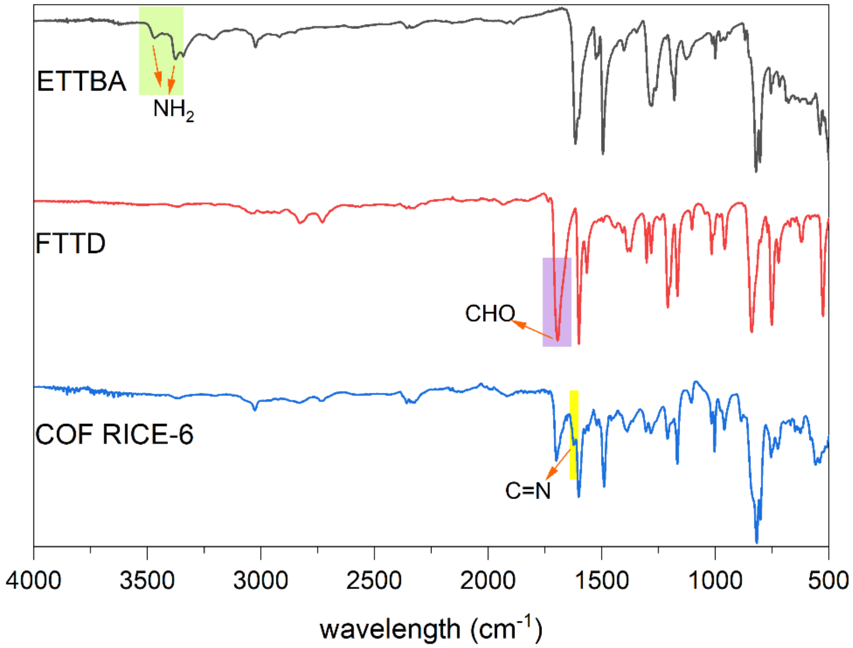

Supplementary Fig. 9. FTIR spectra of COF RICE-6 and its starting monomers.

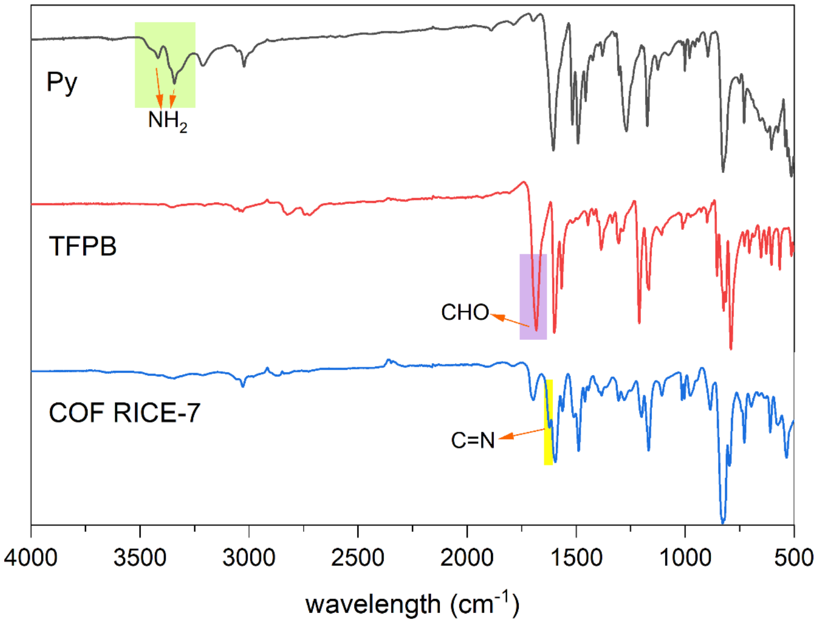

Supplementary Fig. 10. FTIR spectra of COF RICE-7 and its starting monomers.

^{13}C solid-state NMR

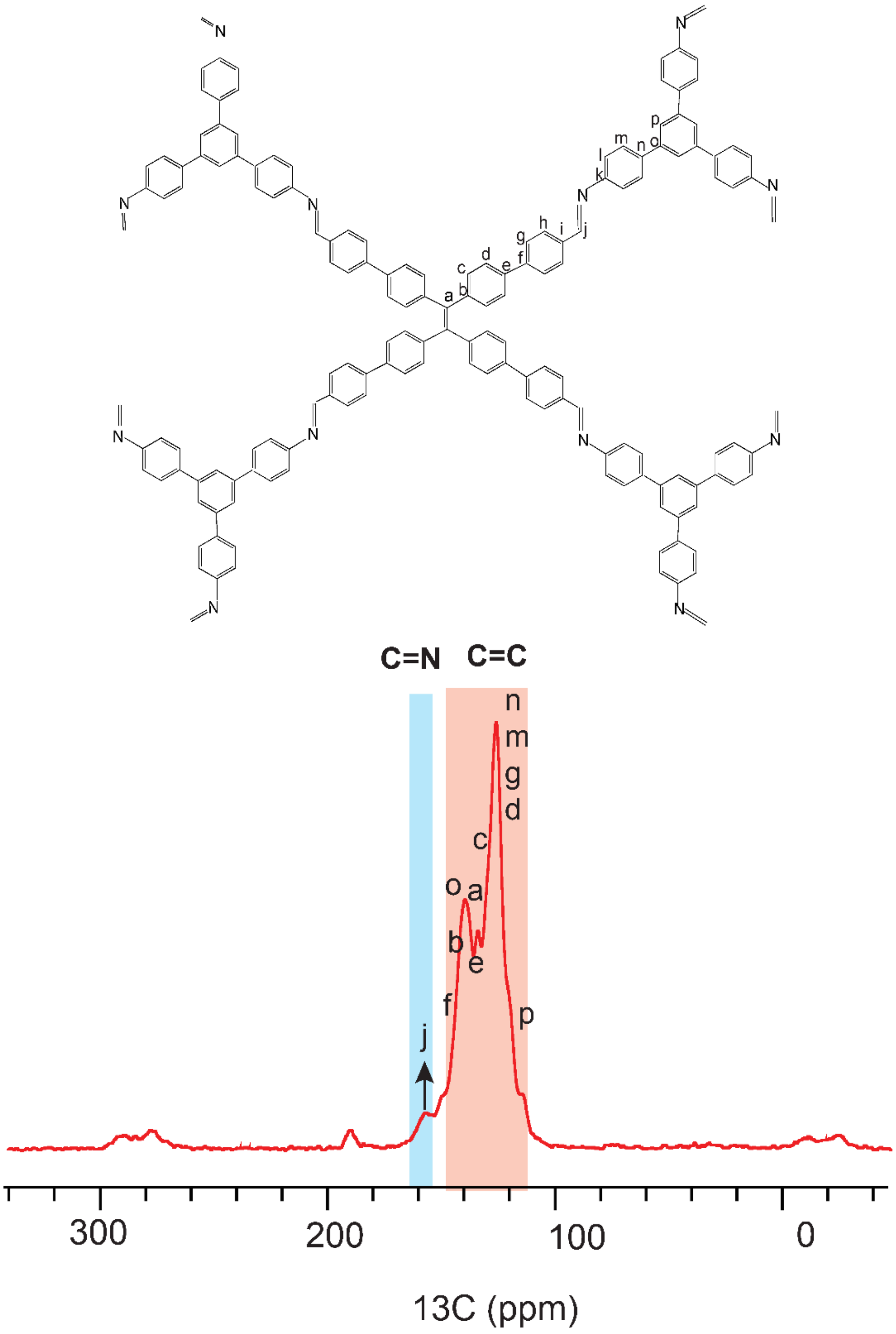

Supplementary Fig. 11. Solid state NMR spectrum of COF RICE-3.

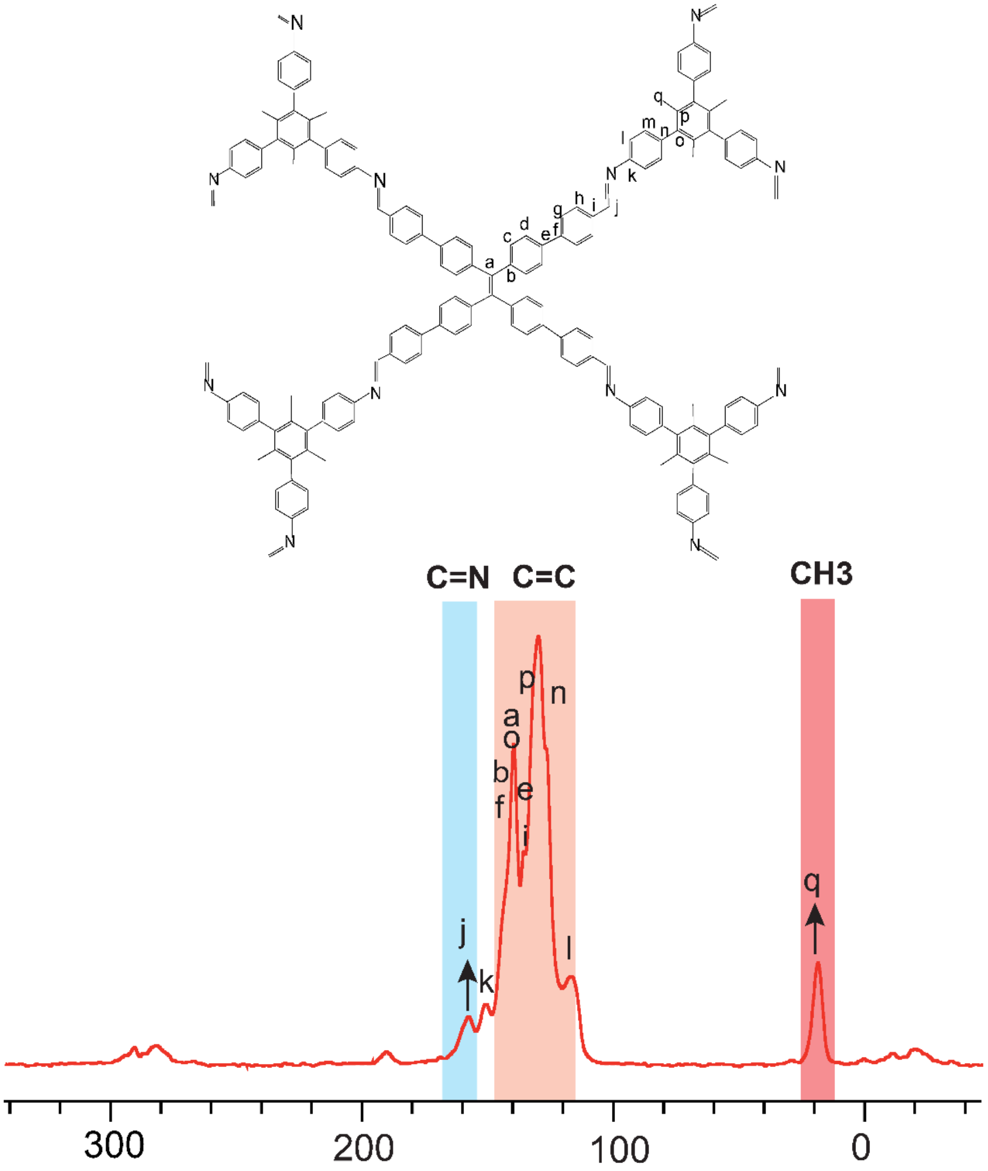

Supplementary Fig. 12. Solid state NMR spectrum of COF RICE-4.

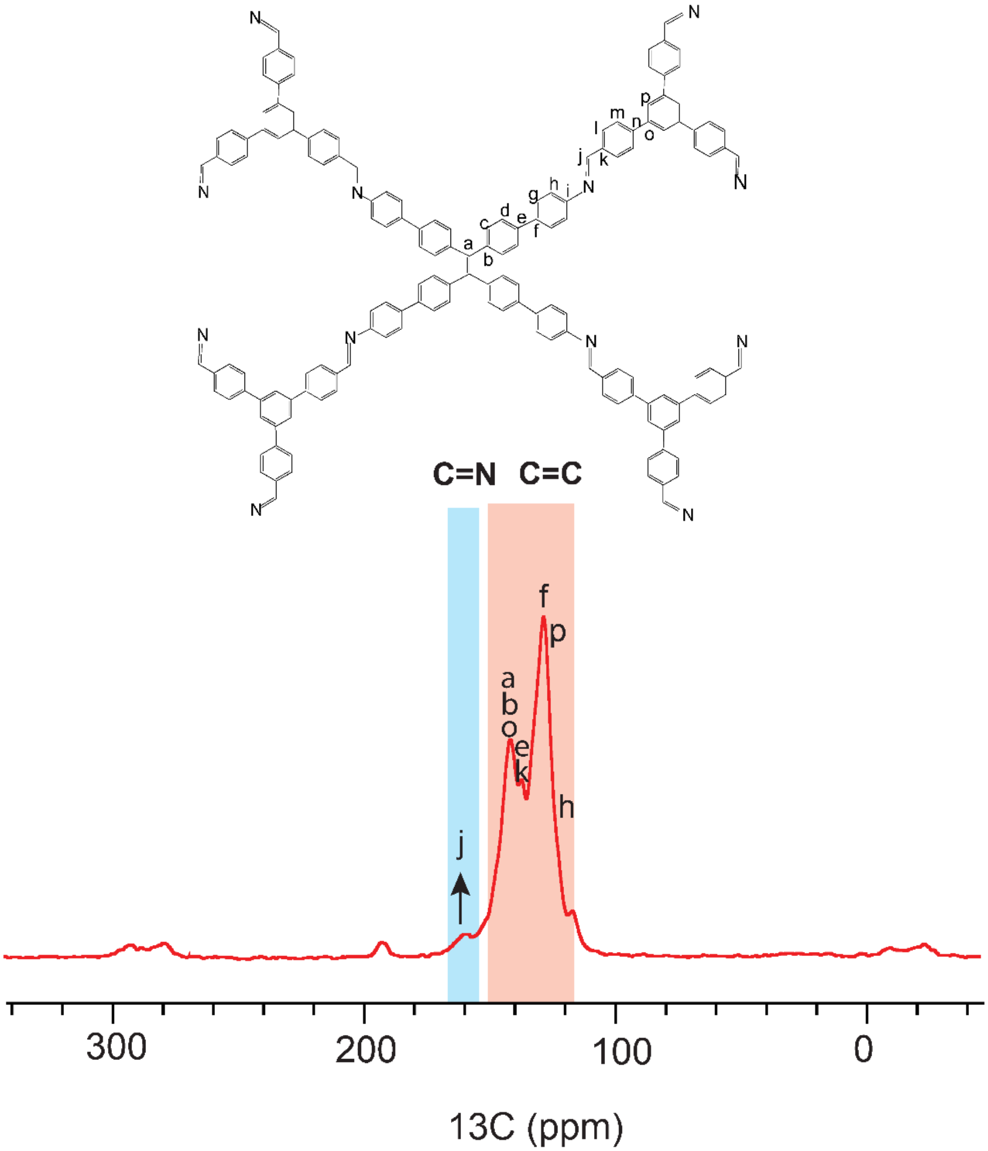

Supplementary Fig. 13. Solid state NMR spectrum of COF RICE-5.

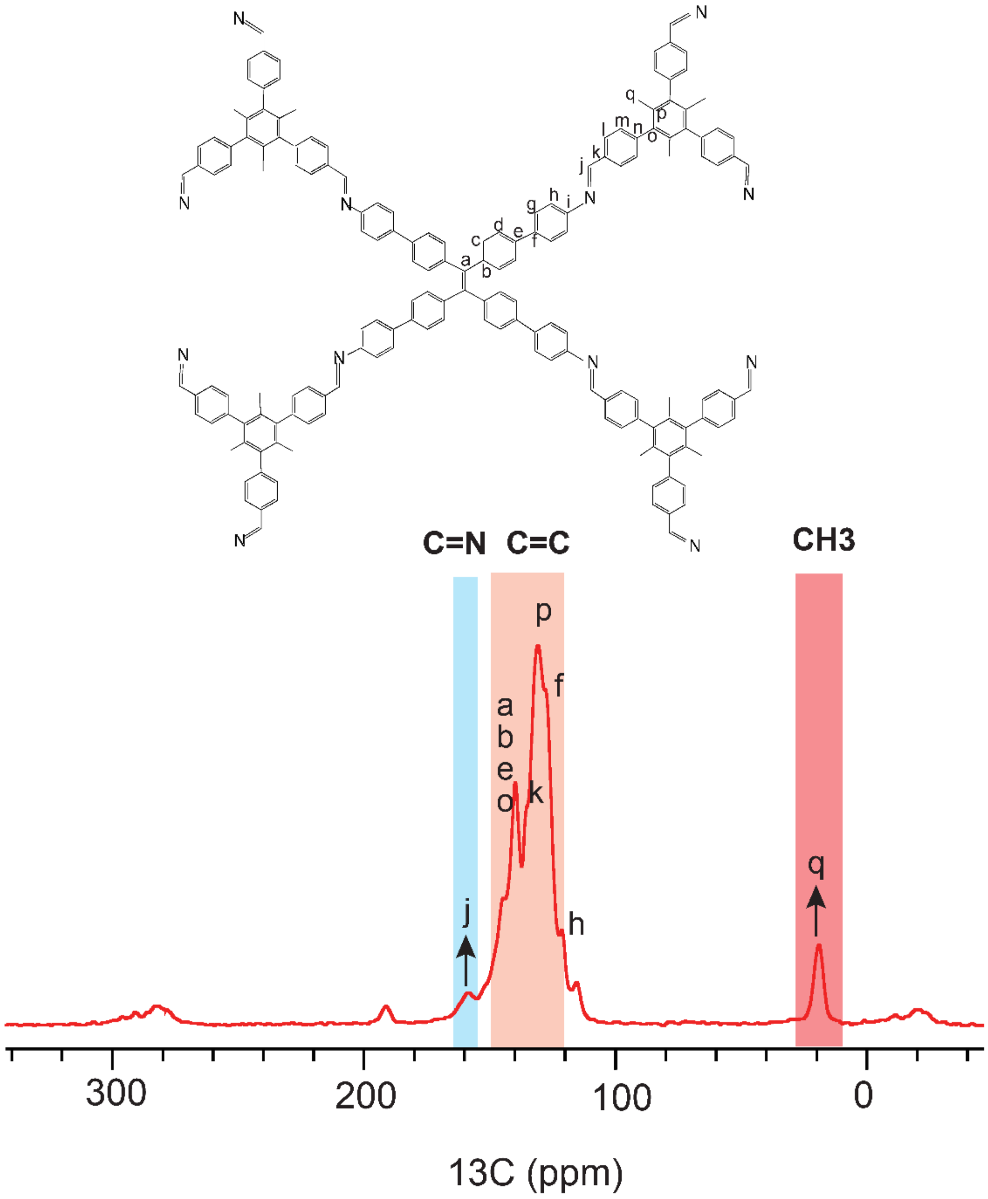

Supplementary Fig. 14. Solid state NMR spectrum of COF RICE-6.

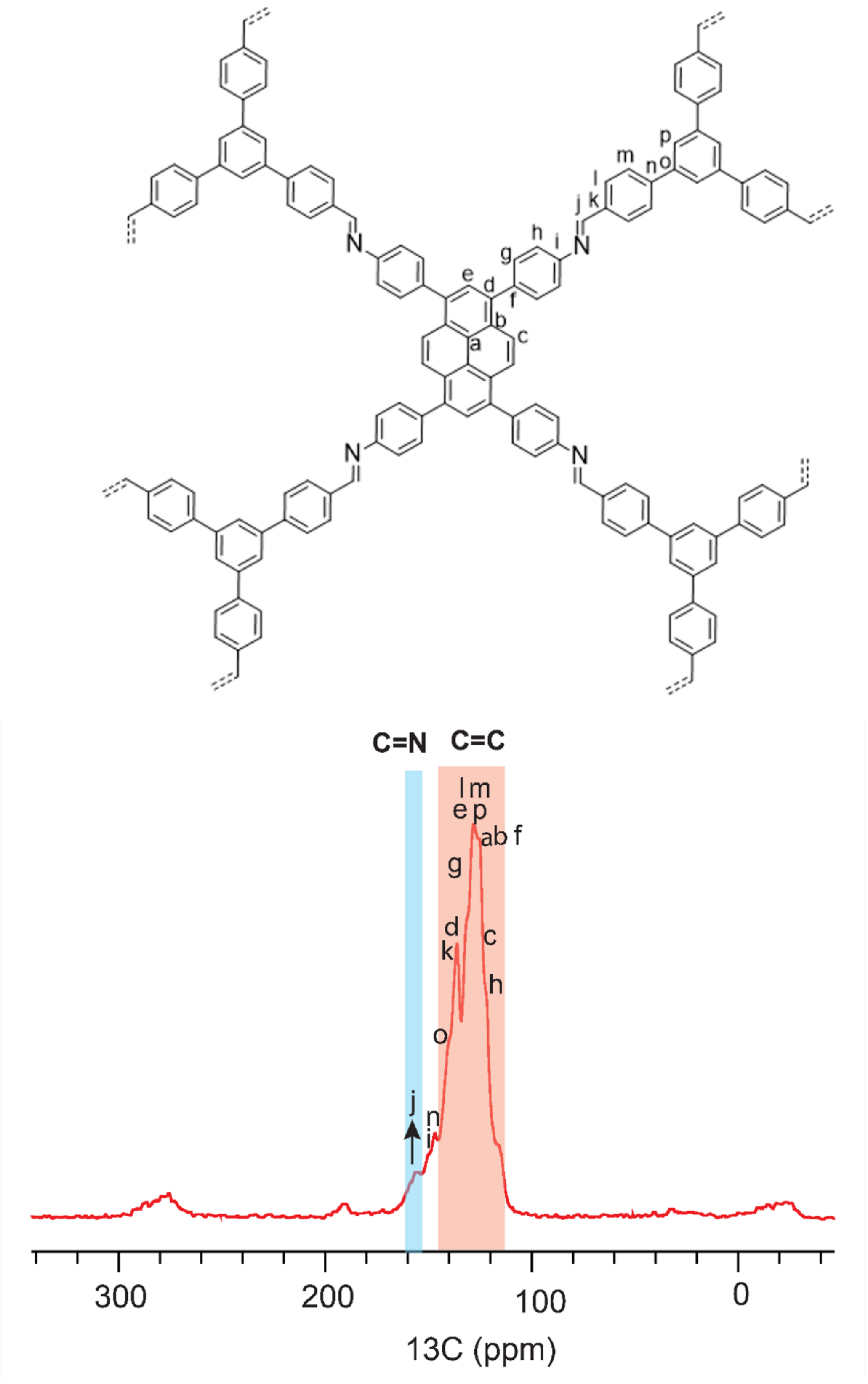

Supplementary Fig. 15. Solid state NMR spectrum of COF RICE-7.

TGA

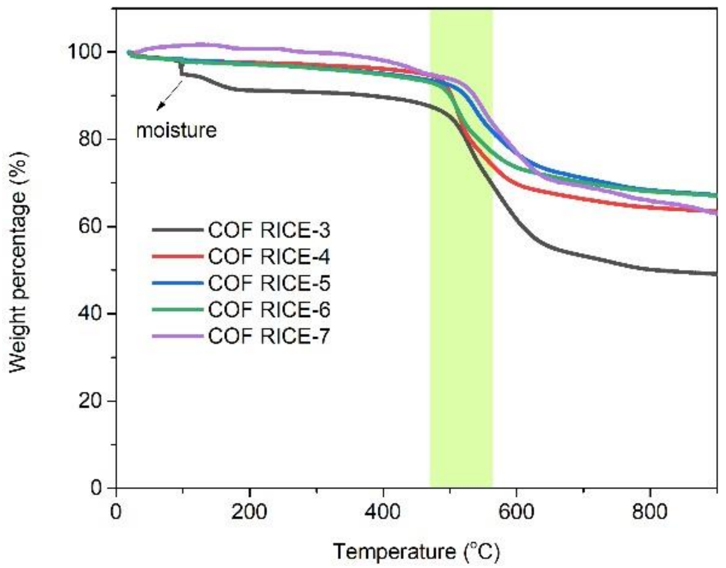

Supplementary Fig. 16. TGA curves for COFs RICE-3/4/5/6/7.

PXRD

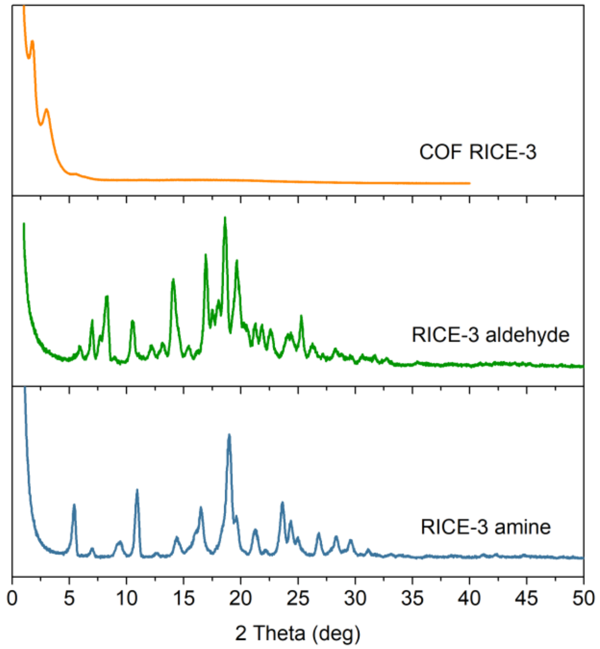

Supplementary Fig. 17. PXRD patterns of COF RICE-3 and their aldehyde and amine monomers.

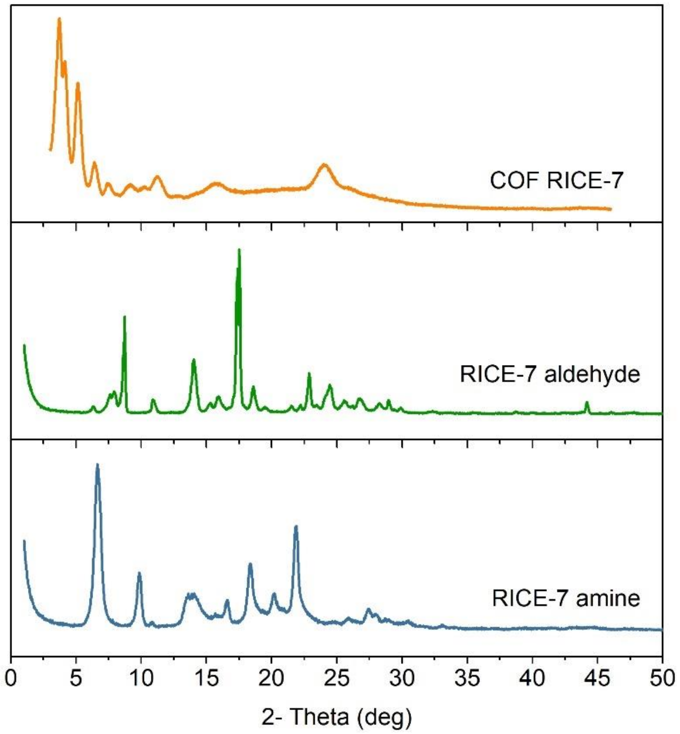

Supplementary Fig. 18. PXRD patterns of COF RICE-7 and their aldehyde and amine monomers.

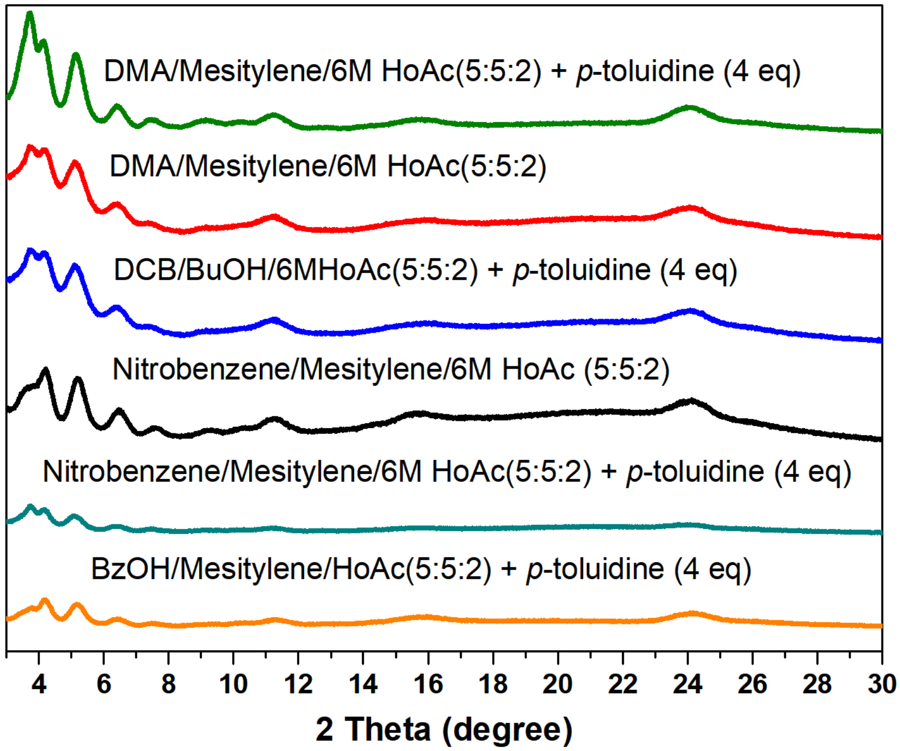

Supplementary Fig. 19. PXRD patterns of COF RICE-7 synthesized using other different conditions

SEM

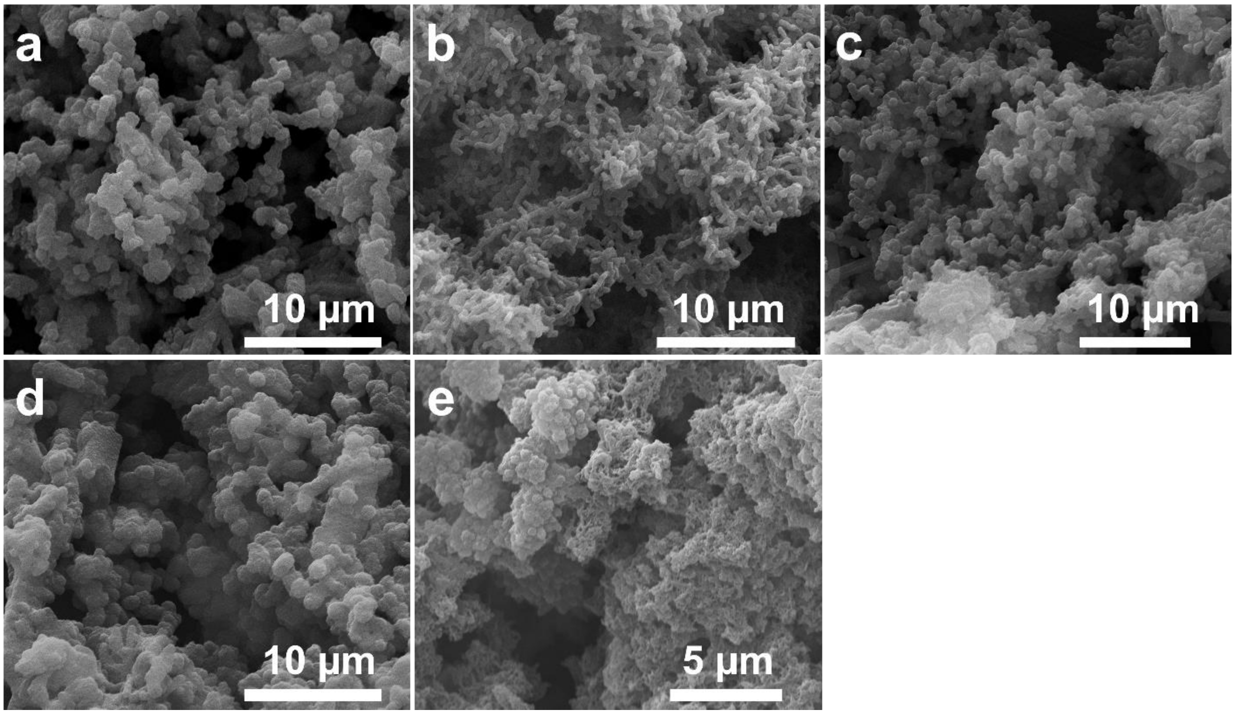

Supplementary Fig. 20. SEM images of COFs (a) RICE-3, (b) RICE-4, (c) RICE-5, (d) RICE-6 and (e) RICE-7.

TEM

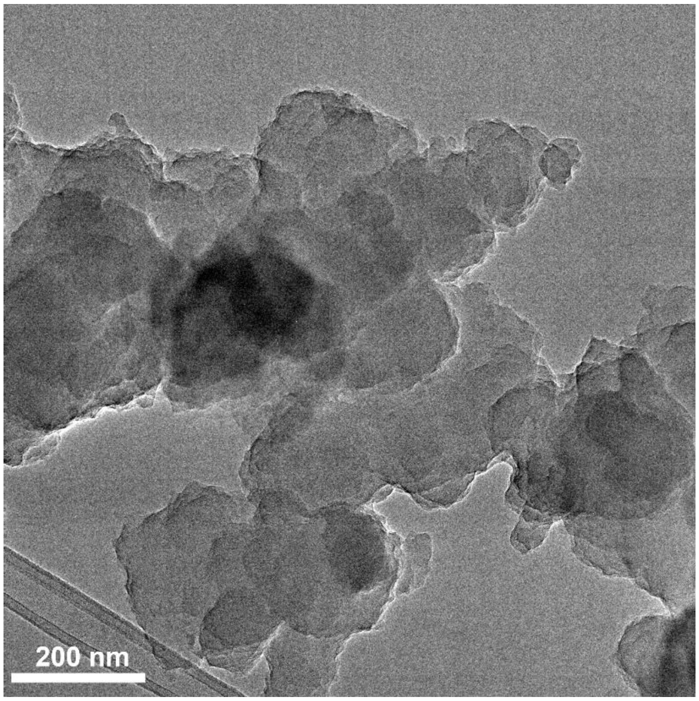

Supplementary Fig. 21. TEM image of COF RICE-3

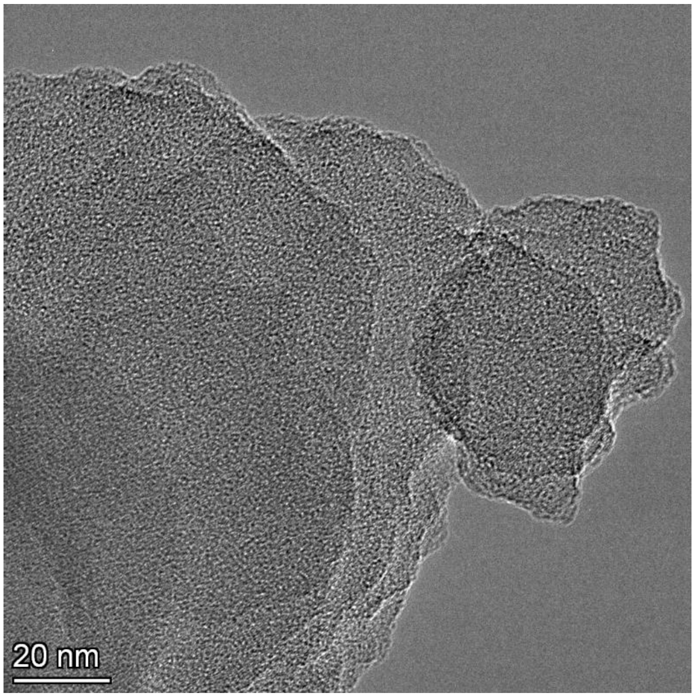

Supplementary Fig. 22. TEM image of COF RICE-3

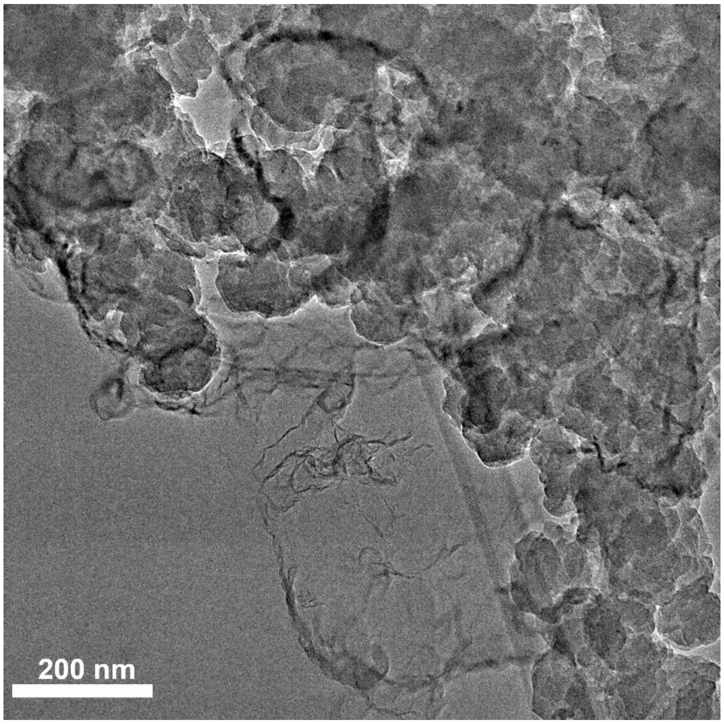

Supplementary Fig. 23. TEM image of COF RICE-4

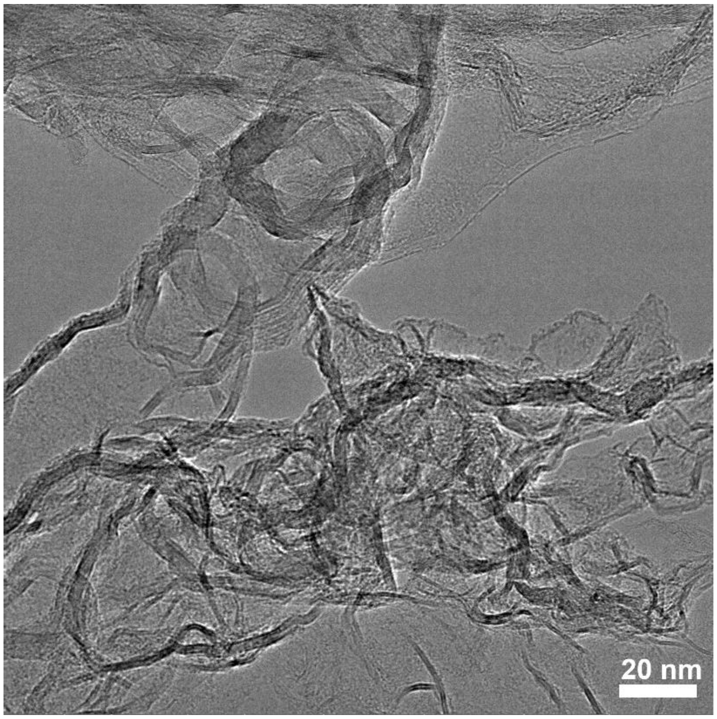

Supplementary Fig. 24. TEM image of COF RICE-4

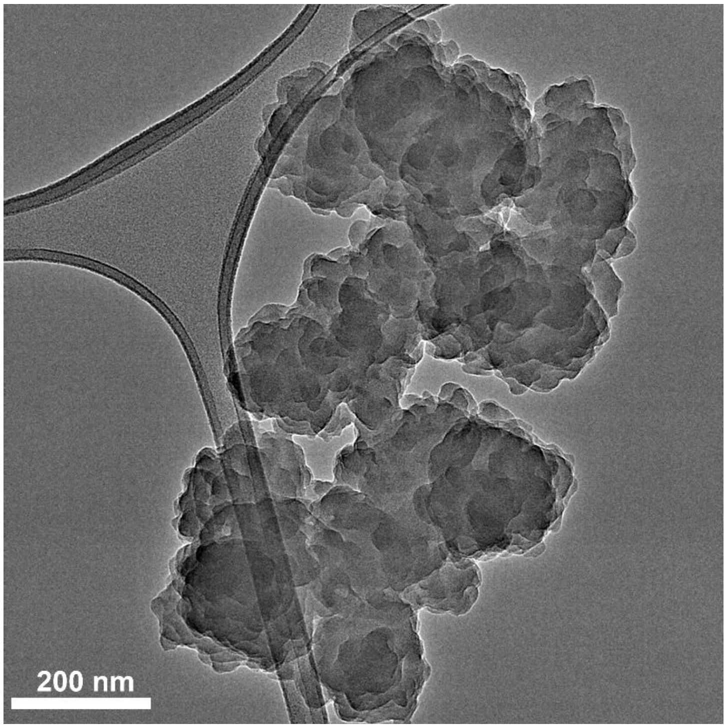

Supplementary Fig. 25. TEM image of COF RICE-5

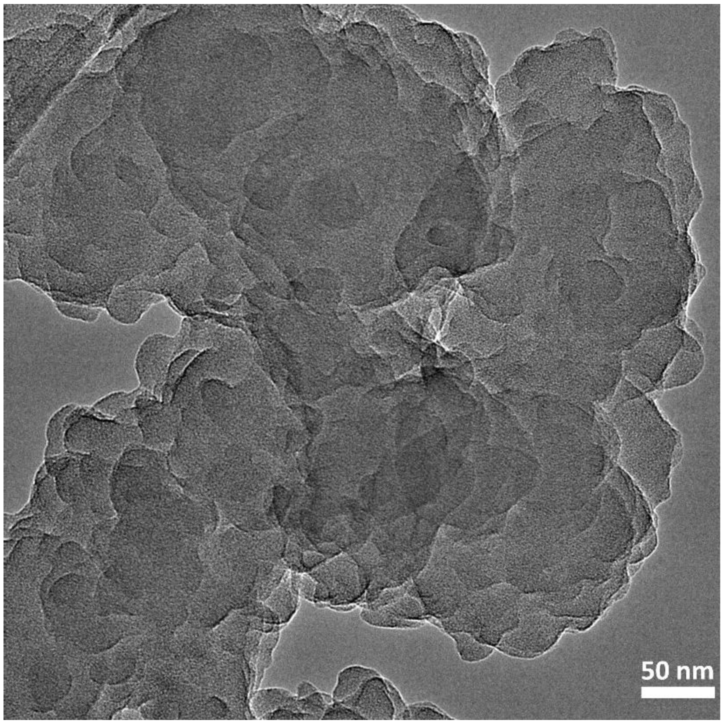

Supplementary Fig. 26. TEM image of COF RICE-5

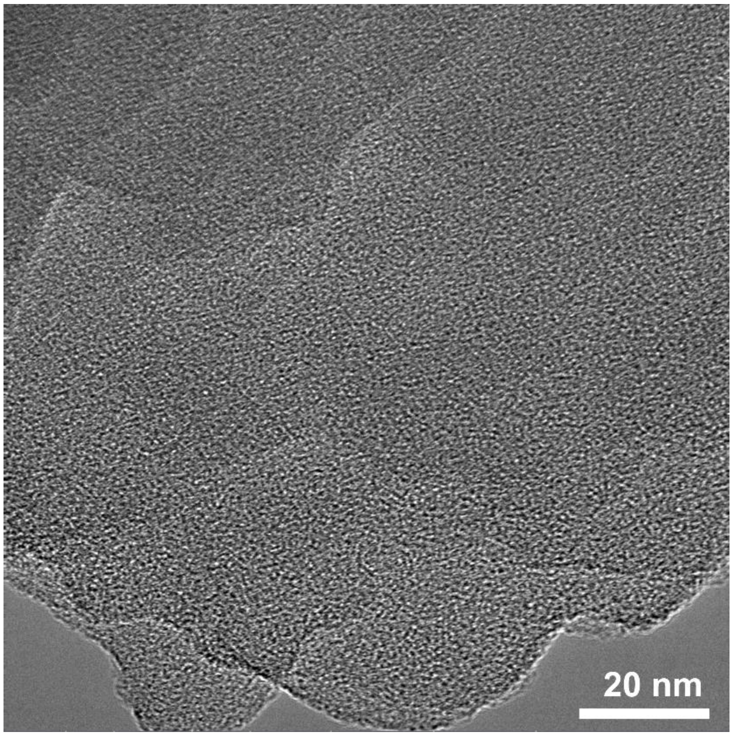

Supplementary Fig. 27. TEM image of COF RICE-5

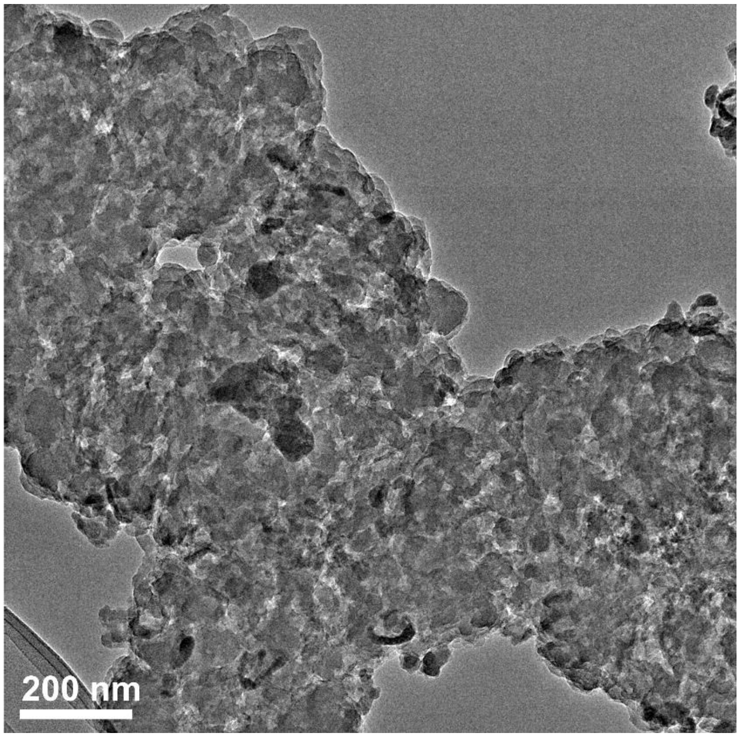

Supplementary Fig. 28. TEM image of COF RICE-6

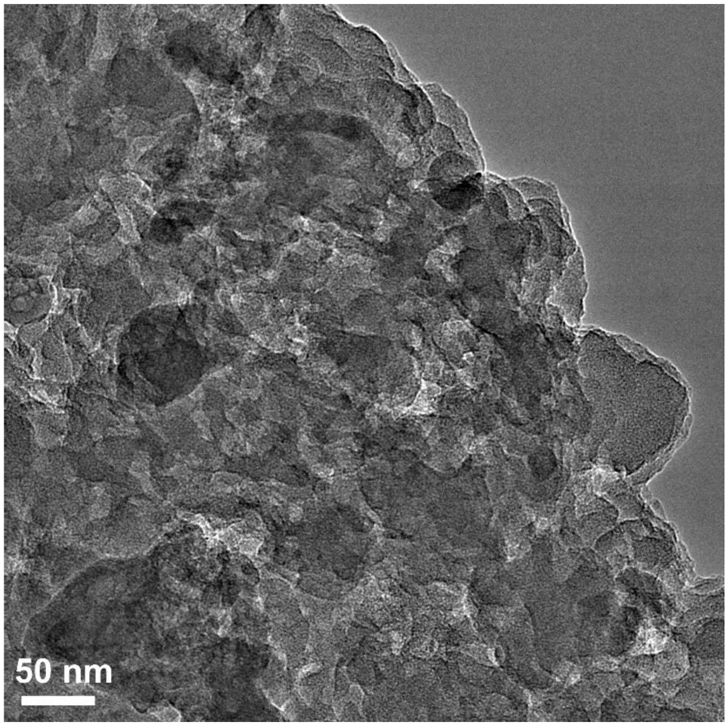

Supplementary Fig. 29. TEM image of COF RICE-6

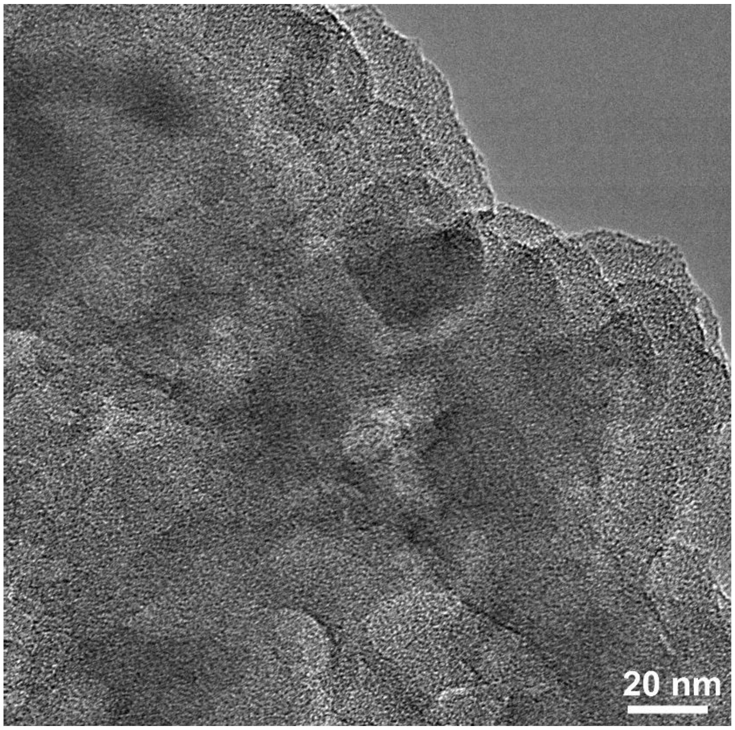

Supplementary Fig. 30. TEM image of COF RICE-6

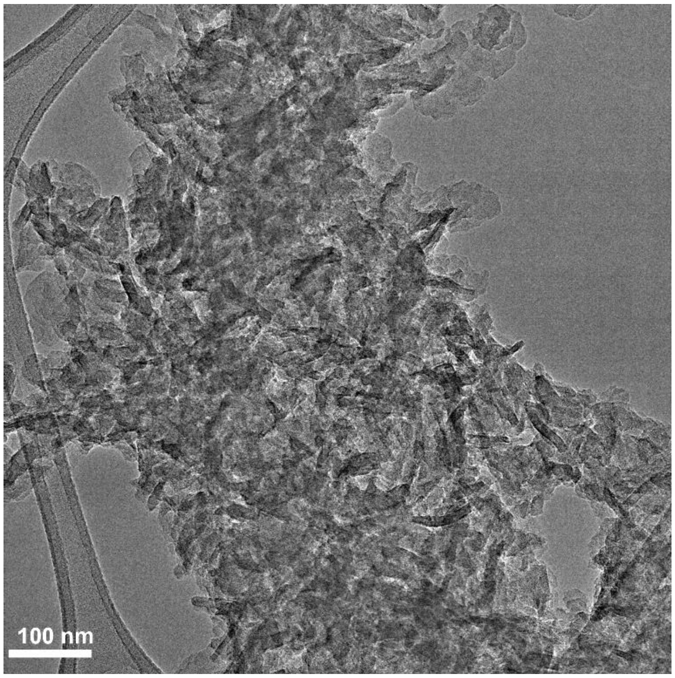

Supplementary Fig. 31. TEM image of COF RICE-7

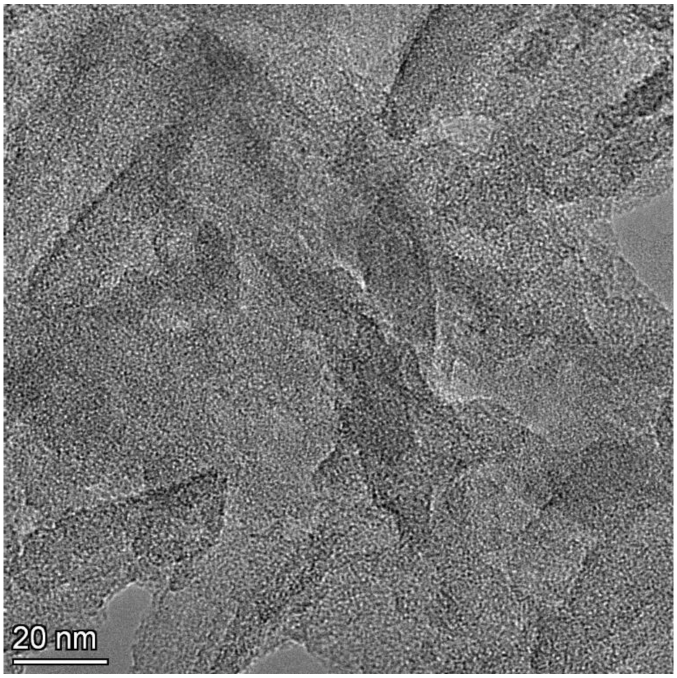

Supplementary Fig. 32. TEM image of COF RICE-7

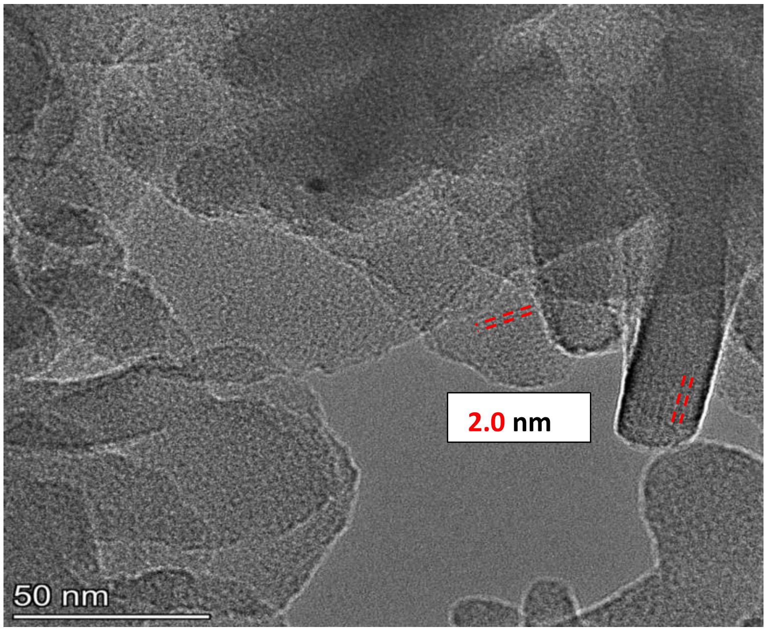

Supplementary Fig. 33. HRTEM images for RICE-7 show crystallites with a periodic channel-like (channel width: 2.0 nm) feature.

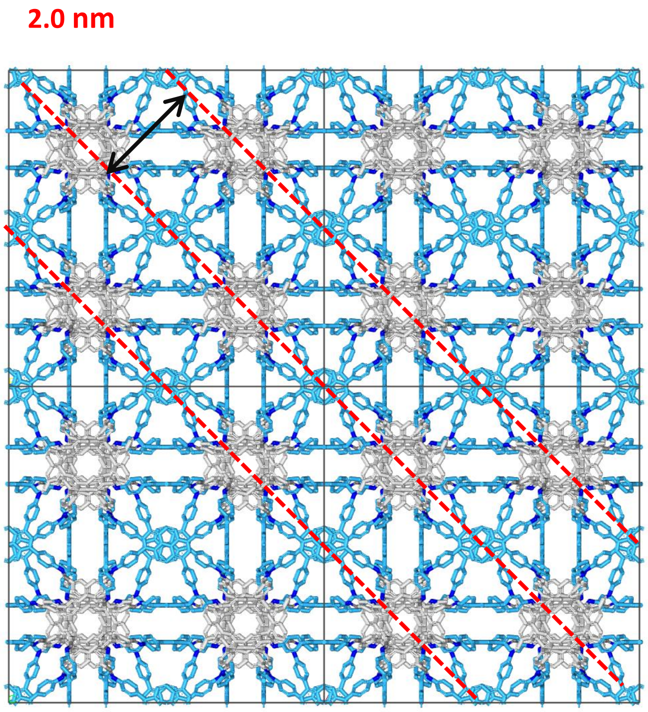

Supplementary Fig. 34. The periodic channel-like feature (channel width: 2.0 nm) in the crystal structure of RICE-7.

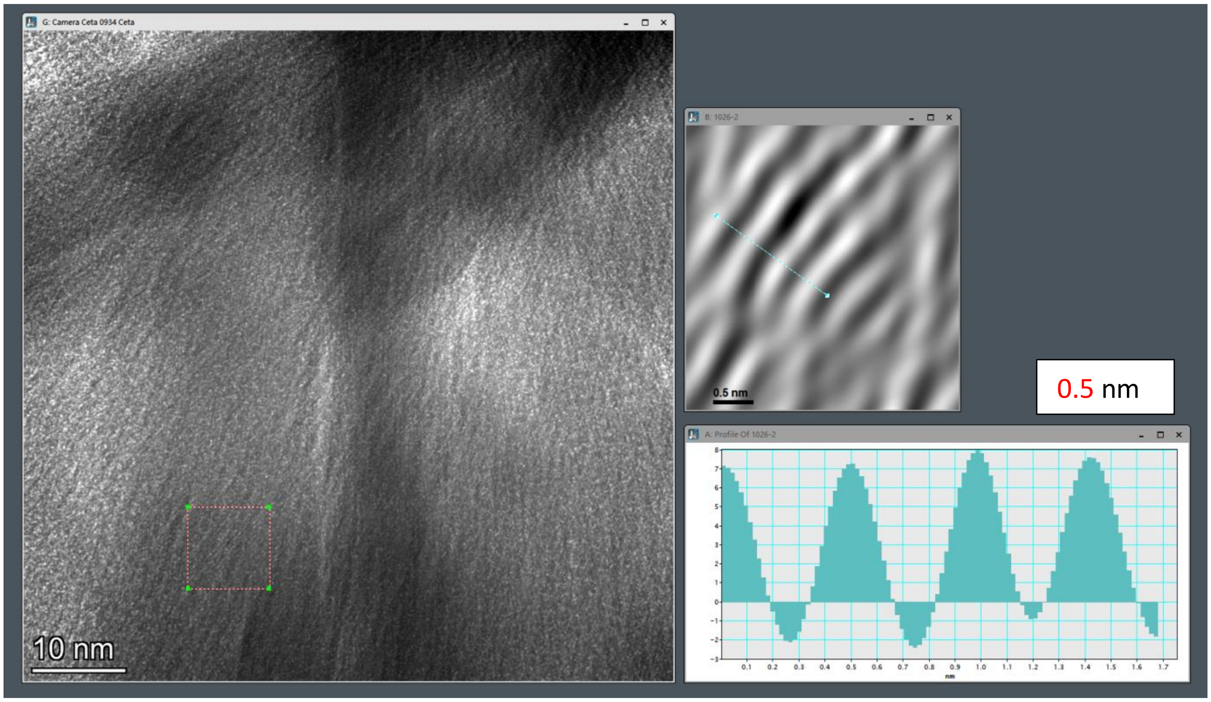

Supplementary Fig. 35. HRTEM images for RICE-7 show a periodic channel-like (channel width: 0.5 nm) feature.

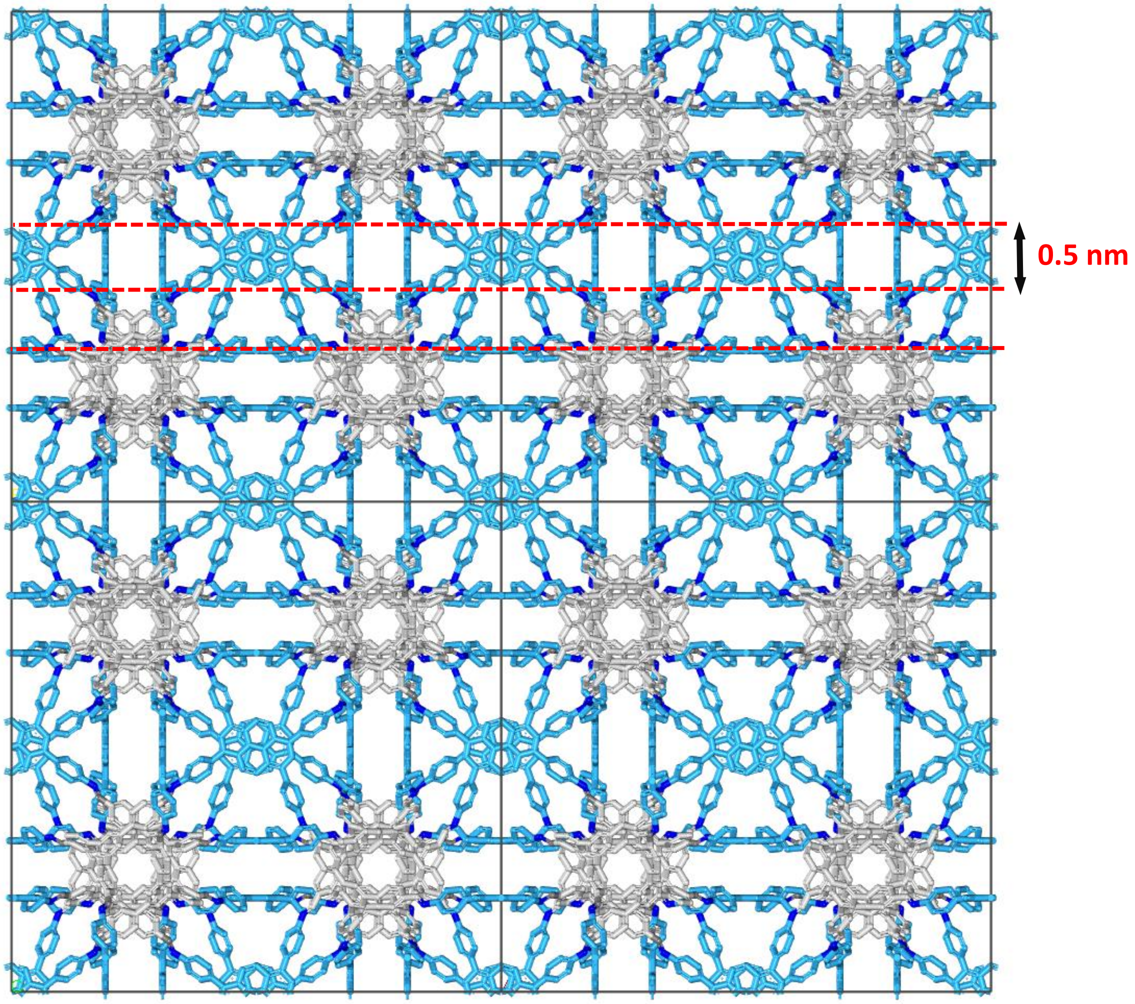

Supplementary Fig. 36. Periodic 0.5 nm channel-like feature in the crystal structure of RICE-7.

Structural modeling and PXRD analysis

Structural modeling for other plausible topologies for RICE-3.

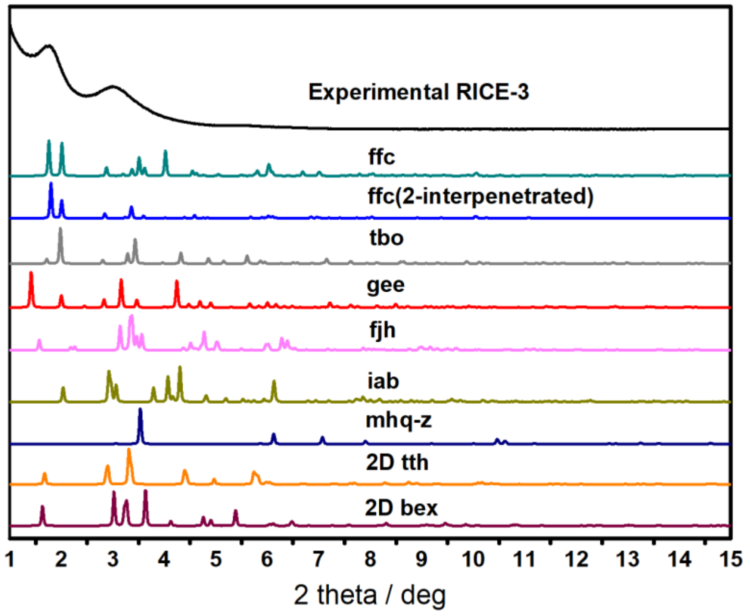

Supplementary Fig. 37. PXRD patterns for other topologies for RICE-3

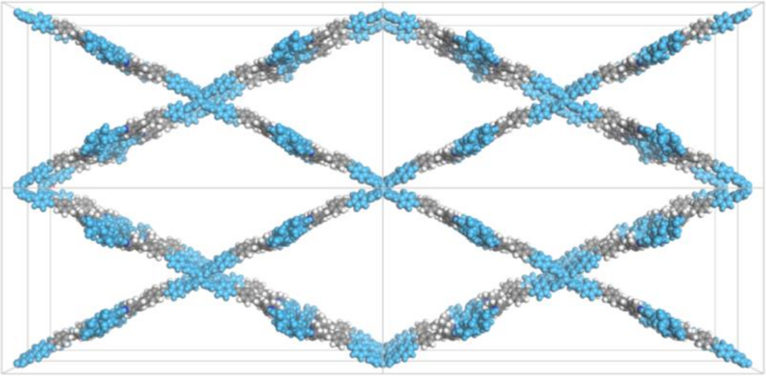

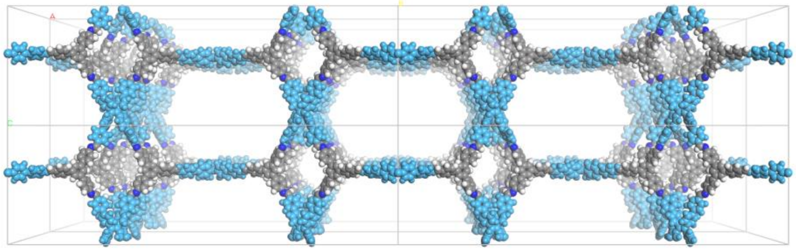

Supplementary Fig. 38. Structure of ffc net for RICE-3

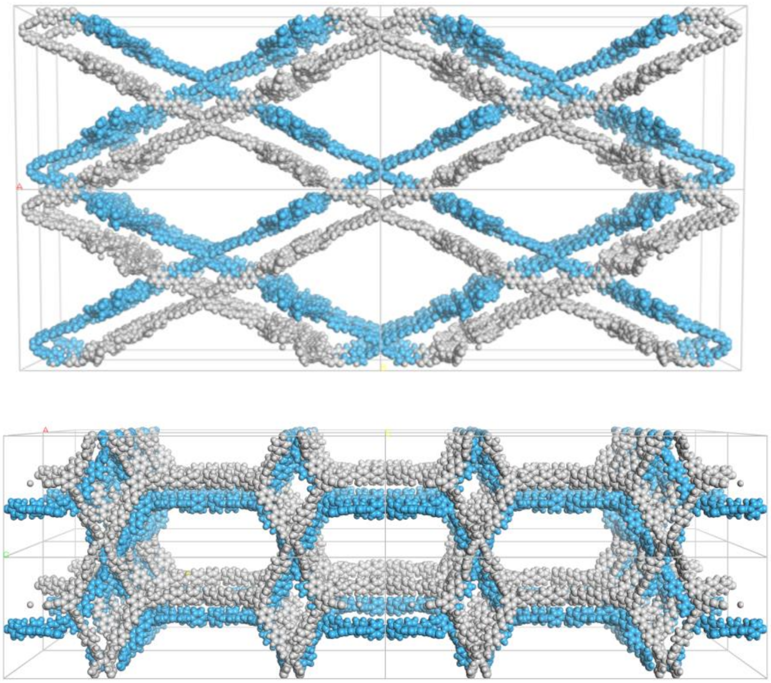

Supplementary Fig. 39. Structure of 2-fold interpenetrated ffc net for RICE-3

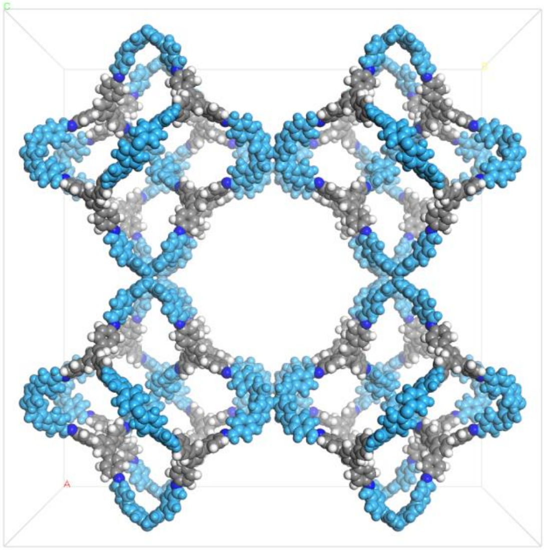

Supplementary Fig. 40. Structure of tbo net for RICE-3

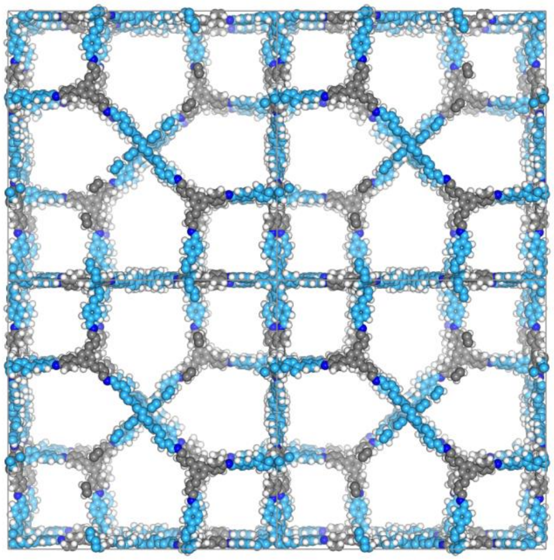

Supplementary Fig. 41. Structure of gee net for RICE-3

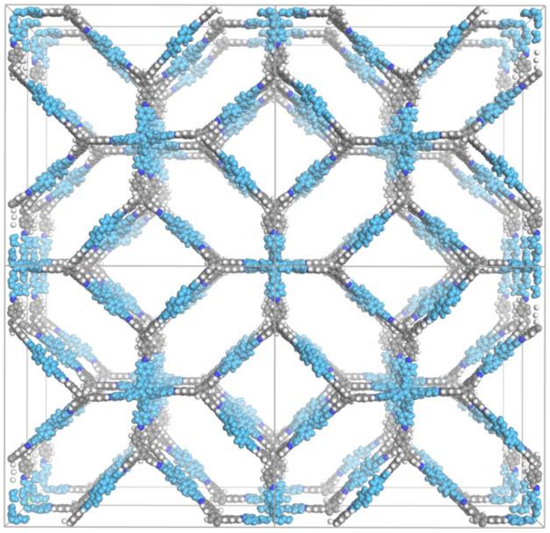

Supplementary Fig. 42. Structure of fjh net for RICE-3

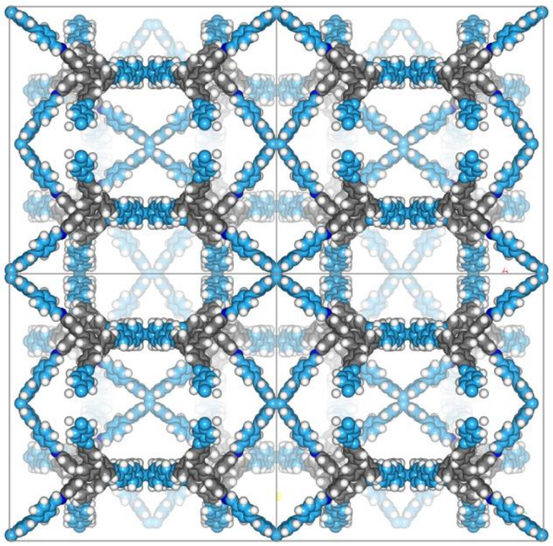

Supplementary Fig. 43. Structure of iab net for RICE-3

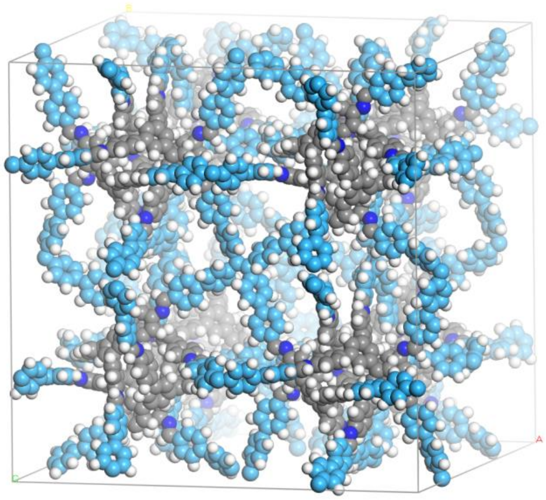

Supplementary Fig. 44. Structure of mhq-z net for RICE-3

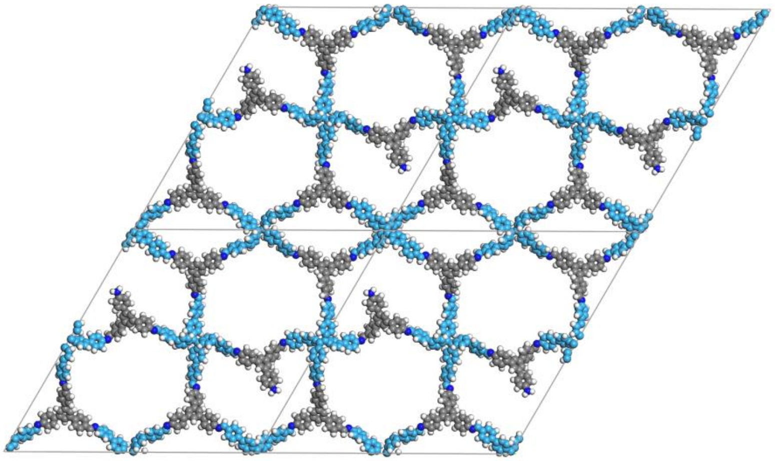

Supplementary Fig. 45. Structure of substoichiometric 2D tth net for RICE-3

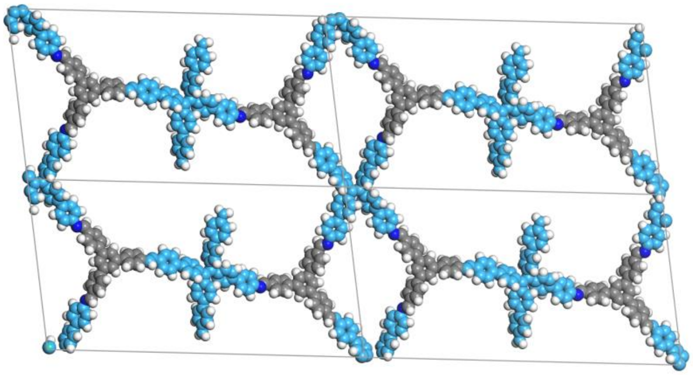

Supplementary Fig. 46. Structure of substoichiometric 2D bex net for RICE-3

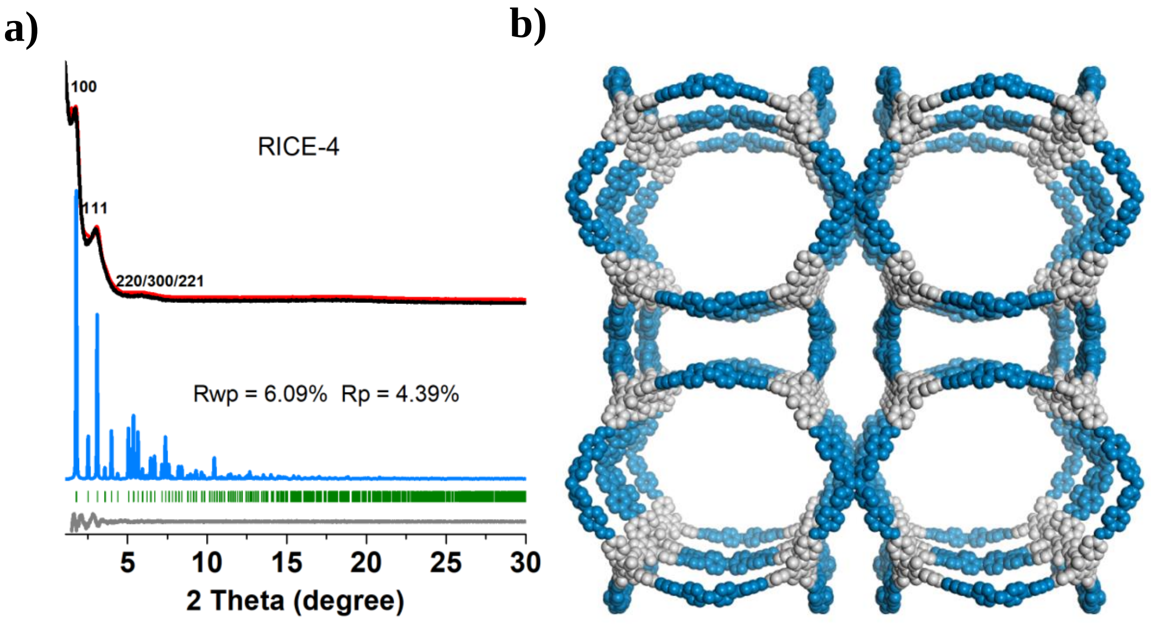

Supplementary Fig. 47. (a) PXRD patterns and refinement result for RICE-4, (b) Structure and pore visualization of RICE-4.

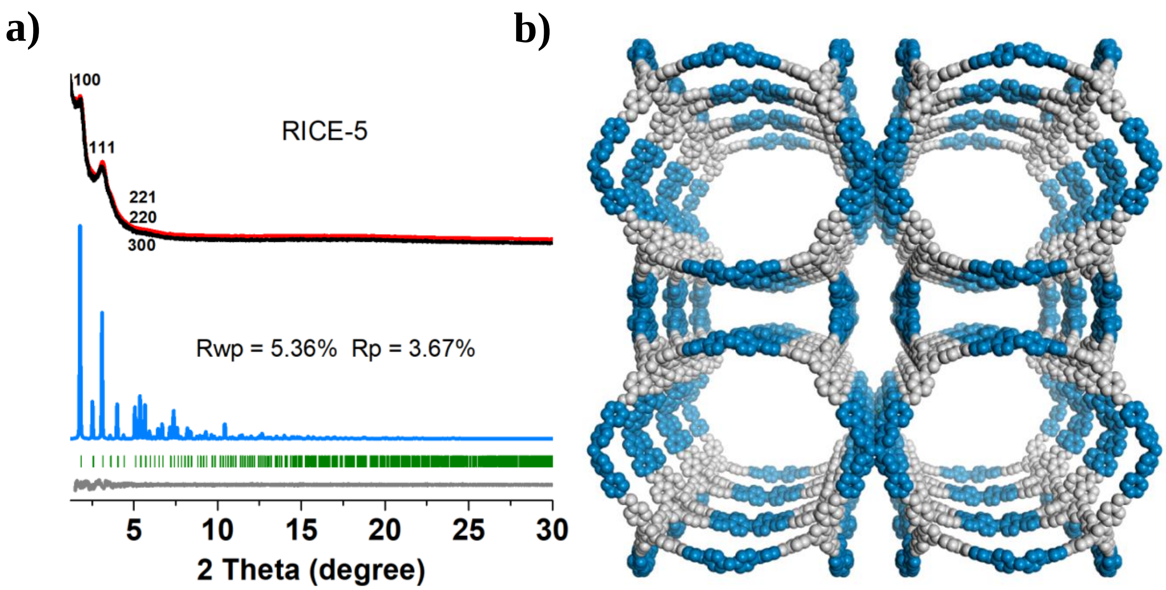

Supplementary Fig. 48. (a) PXRD patterns and refinement result for RICE-5, (b) Structure and pore visualization of RICE-5.

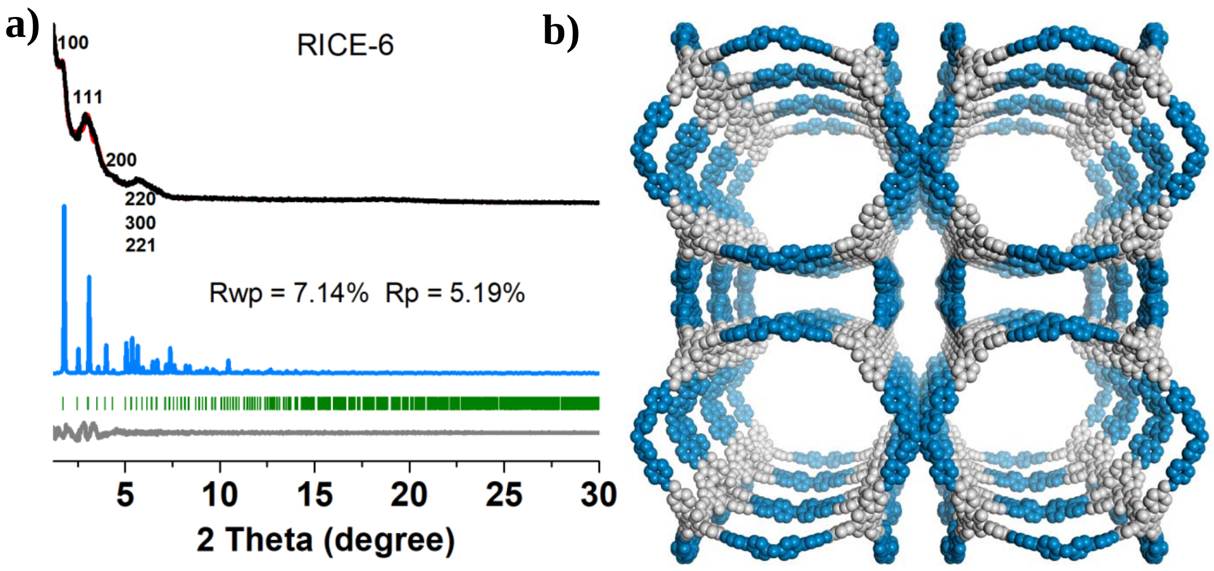

Supplementary Fig. 49. (a) PXRD patterns and refinement result for RICE-6, (b) Structure and pore visualization of RICE-6.

Structural modeling for other plausible topologies for RICE-7.

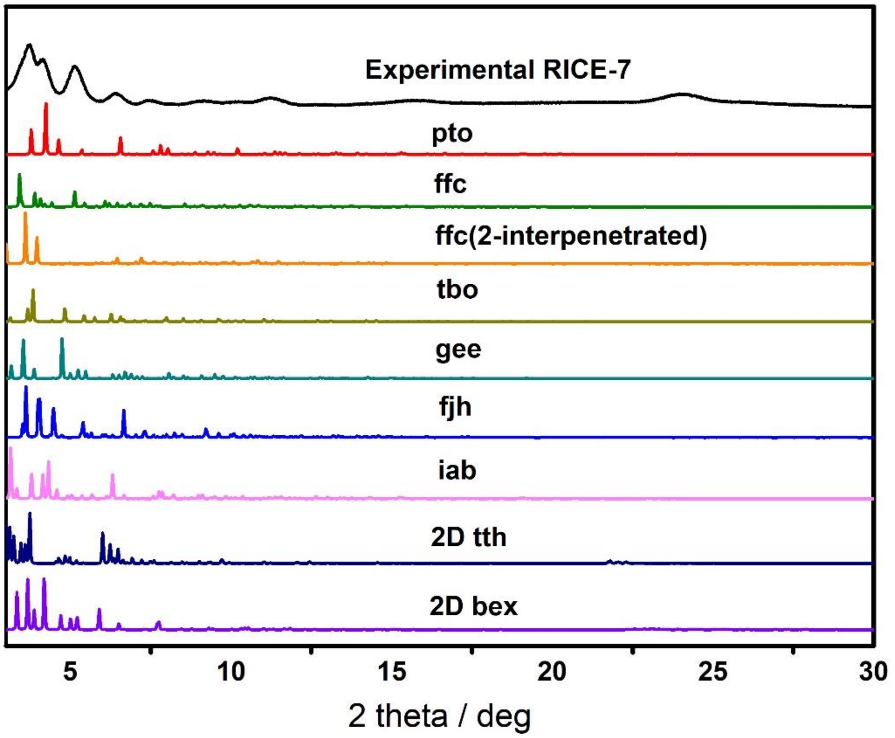

Supplementary Fig. 50. PXRD patterns for other topologies for RICE-7

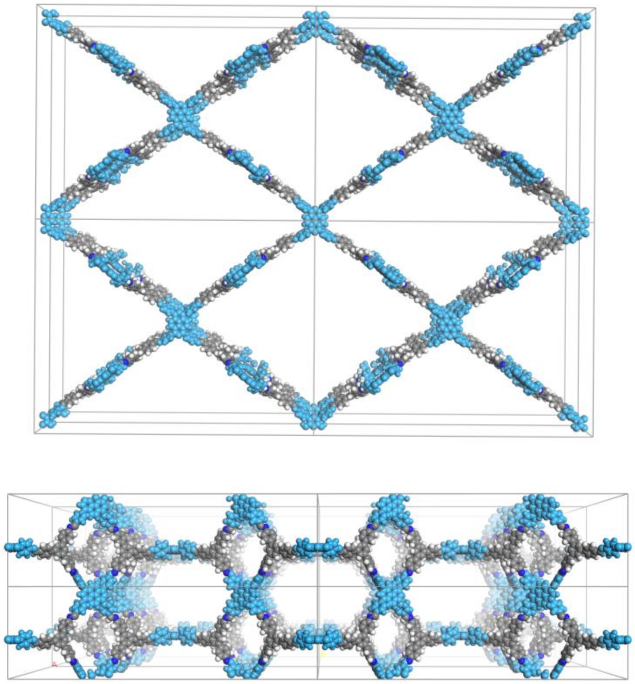

Supplementary Fig. 51. Structure of ffc net for RICE-7

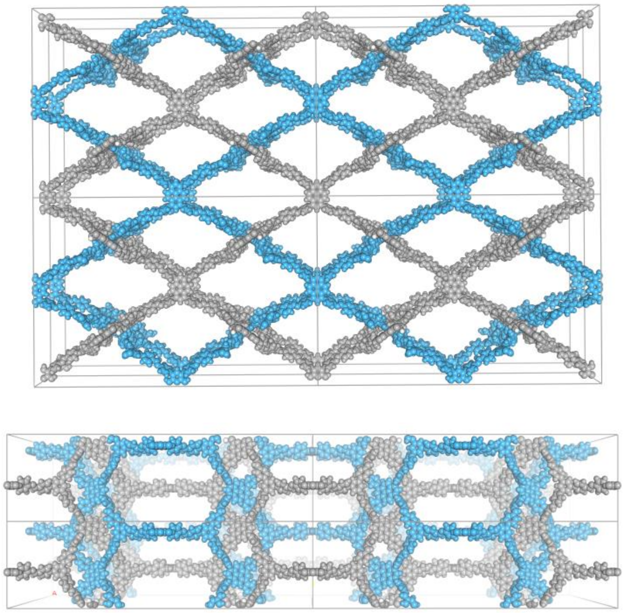

Supplementary Fig. 52. Structure of 2 fold interpenetrated ffc net for RICE-7

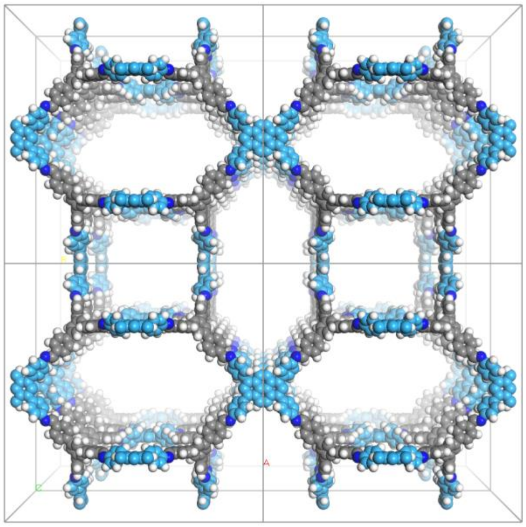

Supplementary Fig. 53. Structure of pto net for RICE-7

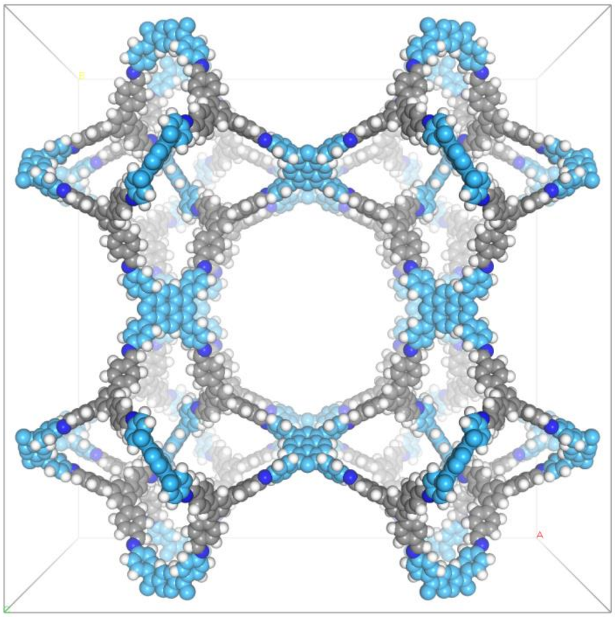

Supplementary Fig. 54. Structure of tbo net for RICE-7

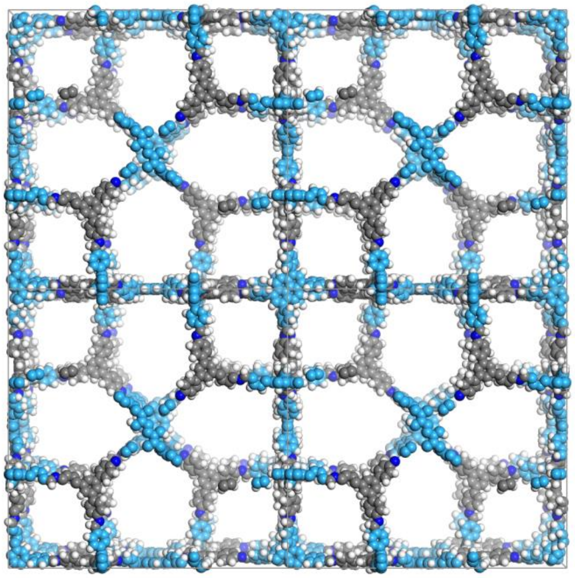

Supplementary Fig. 55. Structure of gee net for RICE-7

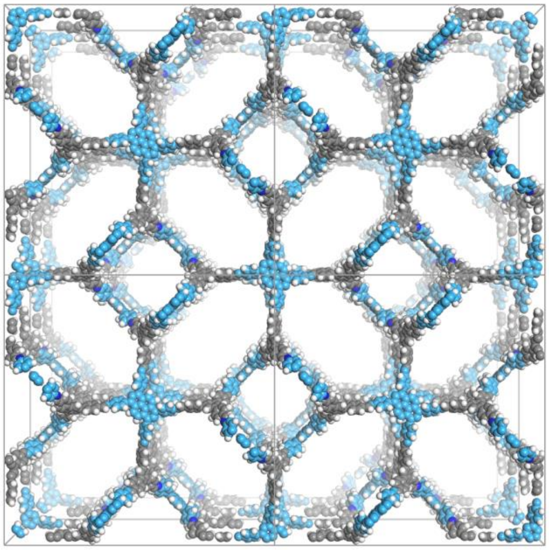

Supplementary Fig. 56. Structure of fjh net for RICE-7

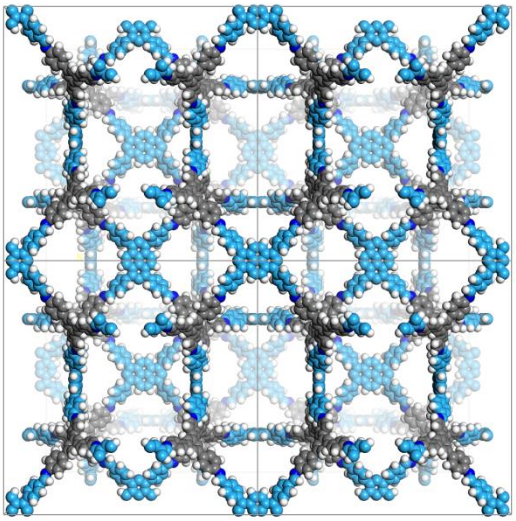

Supplementary Fig. 57. Structure of iab net for RICE-7

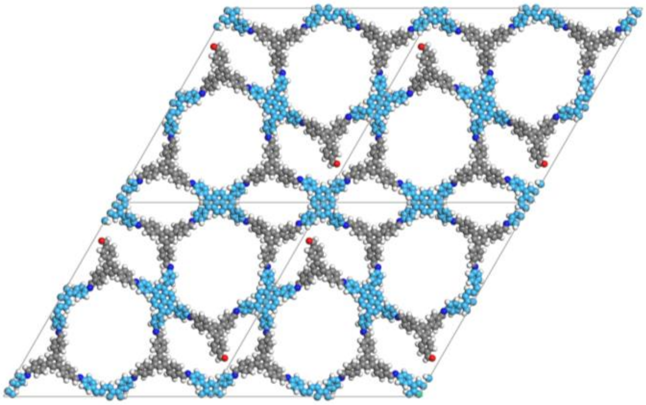

Supplementary Fig. 58. Structure of substoichiometric 2D tth net for RICE-7

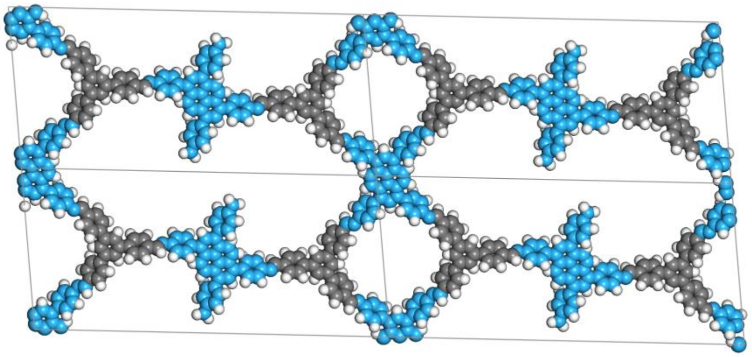

Supplementary Fig. 59. Structure of substoichiometric 2D bex net for RICE-7

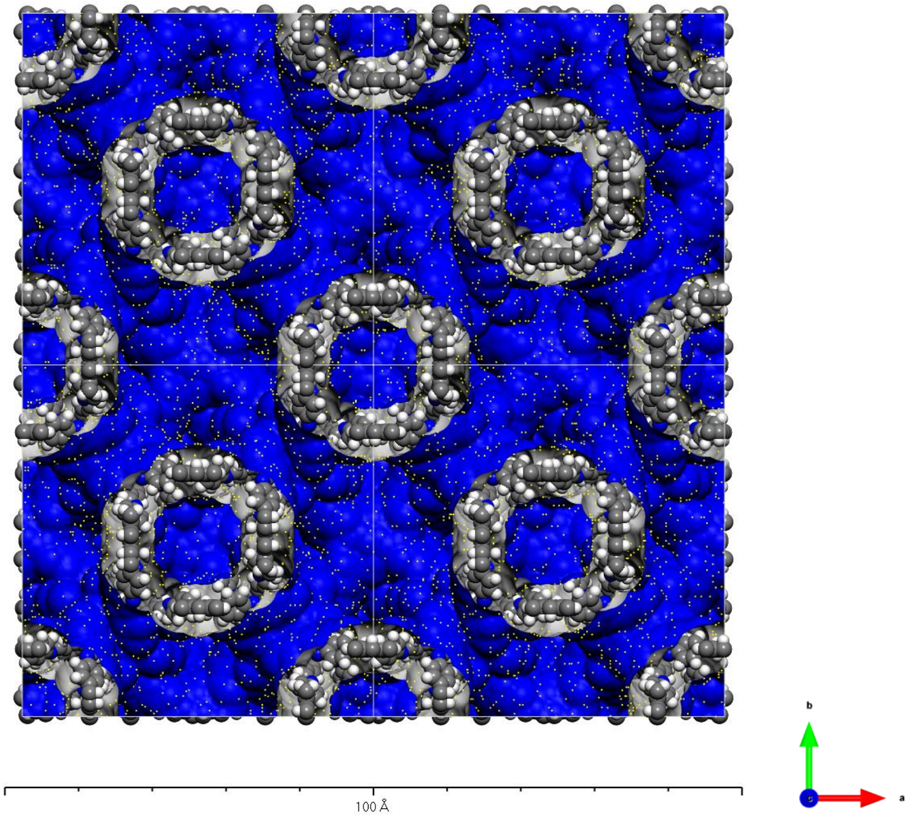

Supplementary Fig. 60. Pore visualization from 001/100 facet for RICE-7.

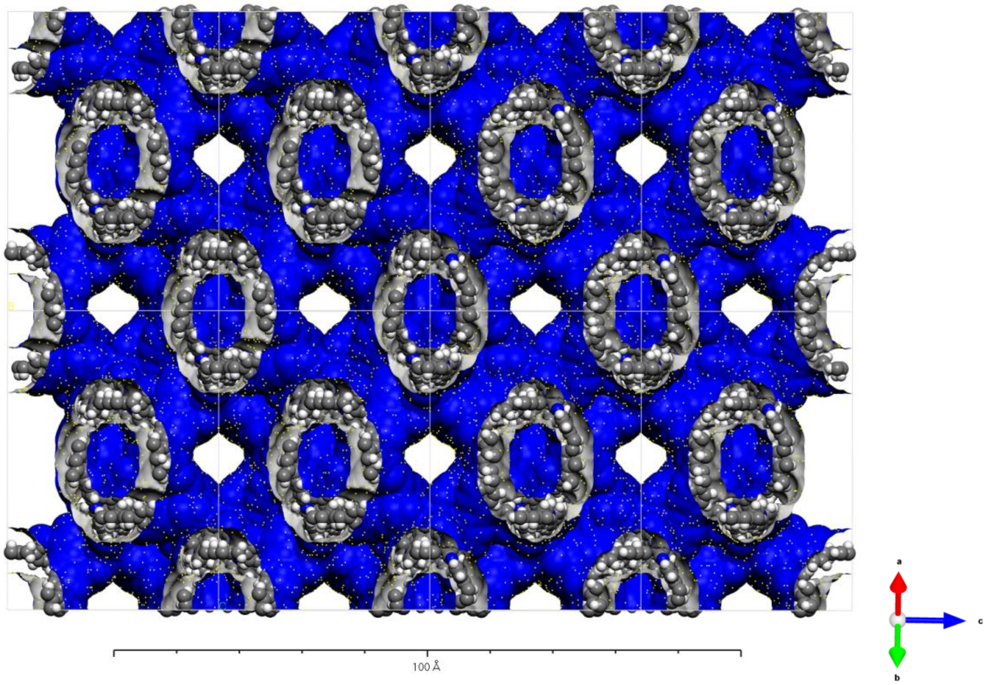

Supplementary Fig. 61. Pore visualization from the 110 facet for RICE-7.

Single crystal structures of monomers and conformational analyses.

We found that the single crystal structures of molecular model compounds for RICE-6/7 (TMTP and PBAD, Supplementary Figs. 62/63) had been reported and available in CCDC database (CCDC identifier: 2027484 for TMTP and 1503281 for PBAD). By utilizing these crystal structures, we were able to further analyze their conformations. The dihedral angles between units of TMTP and PBAD were found to be 75−90°, which were nearly identical to that of our proposed pto and mhq-z topologies for RICE-6/7 (Supplementary Figs. 62/63).

(a)

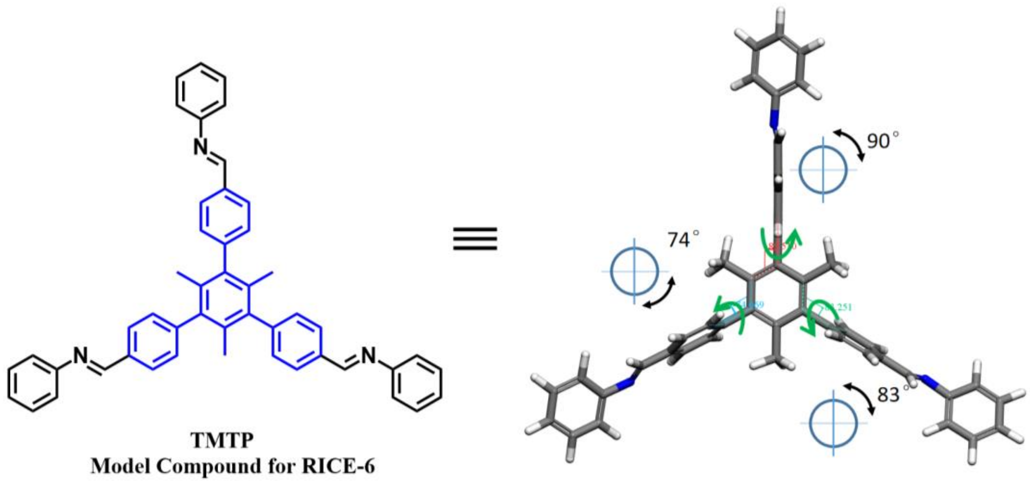

(b)

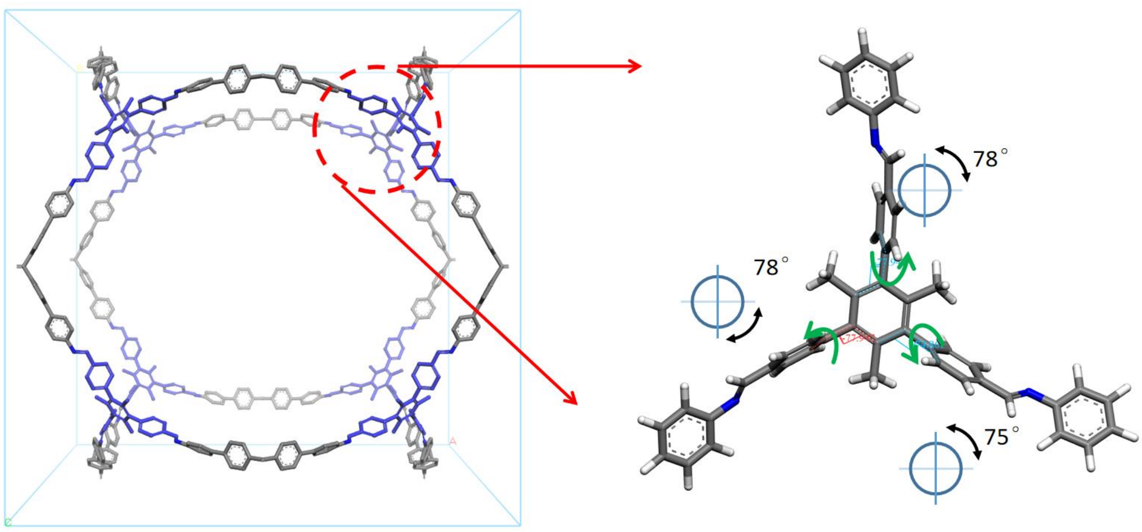

Supplementary Fig. 62. (a) Single-crystal X-ray structure of the model compound for RICE-6 (**TMTP**) with dihedral angles of 74−90°. (b) Dihedral angles of building units in simulated pto topology of RICE-6.

(a)

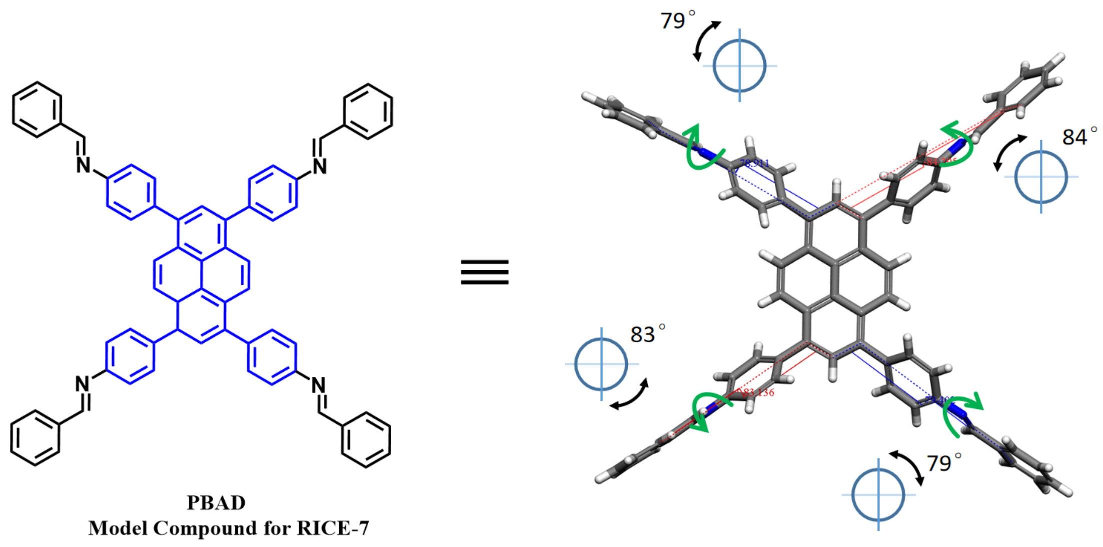

(b)

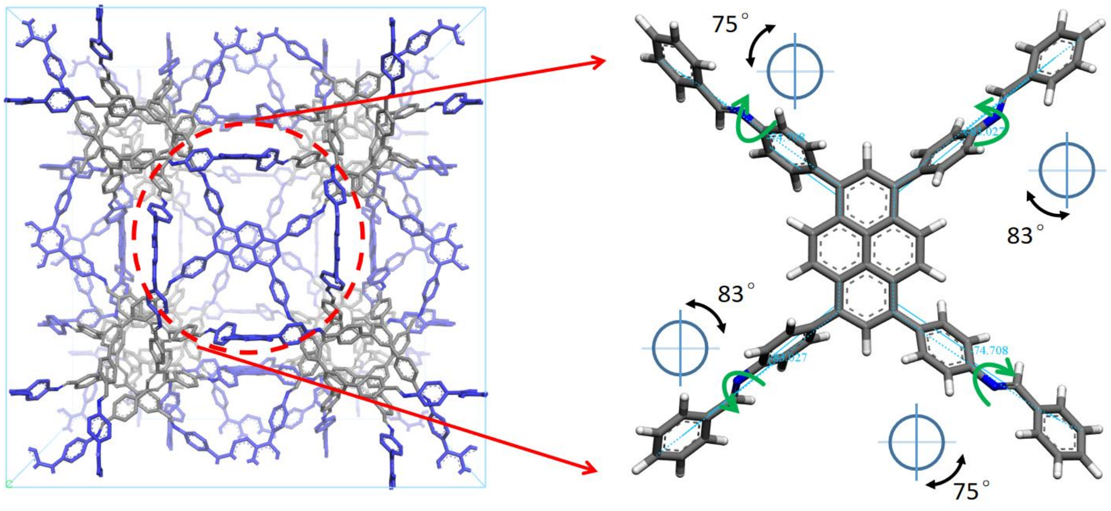

Supplementary Fig. 63. (a) Single-crystal X-ray structure of the model compound for RICE-7 (**PBAD**) with dihedral angles of 75−85°. (b) Dihedral angles of building units in simulated mhq-z topology of RICE-7.

Nitrogen Sorption Measurements

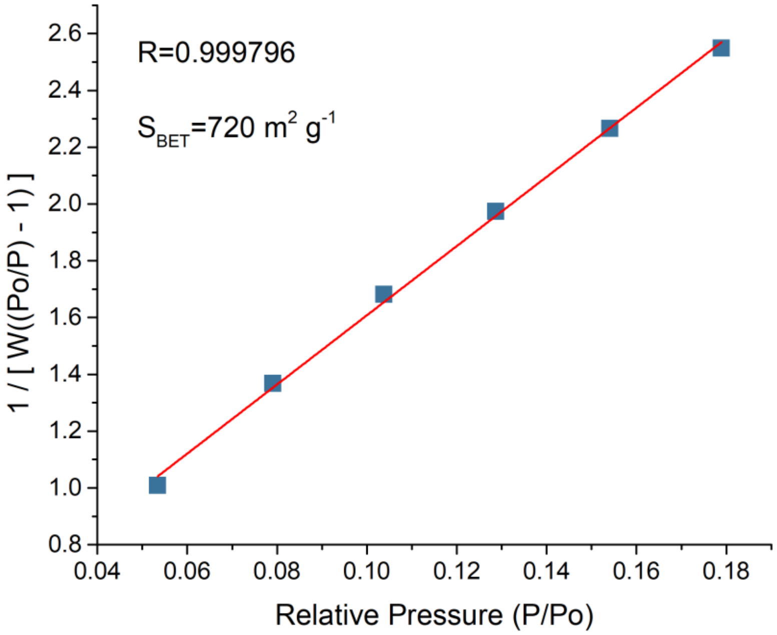

Supplementary Fig. 64. BET linear fitting plot for COF RICE-3.

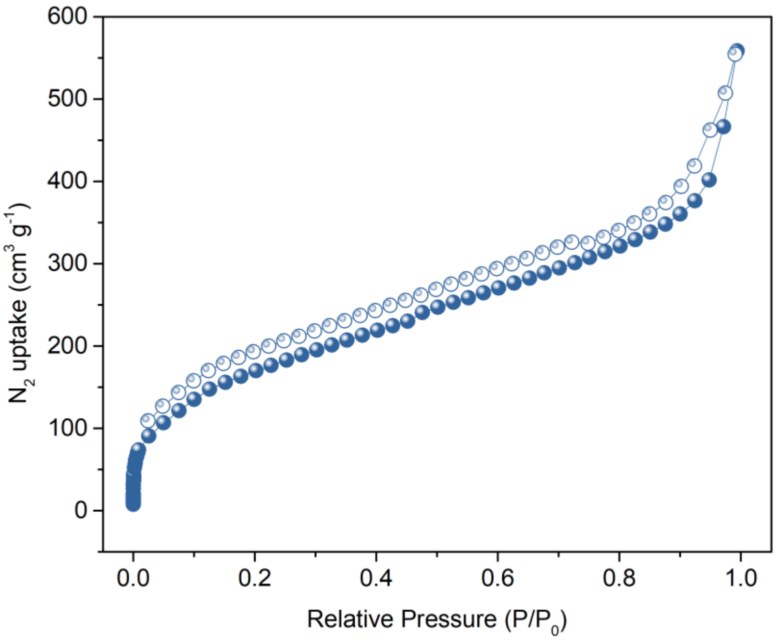

Supplementary Fig. 65. Nitrogen sorption isotherm of COF RICE-4

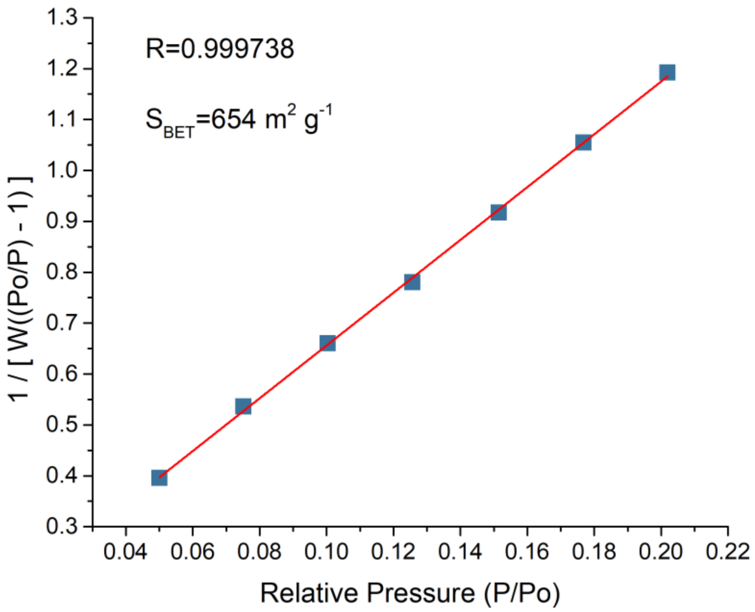

Supplementary Fig. 66. BET linear fitting plot for COF RICE-4.

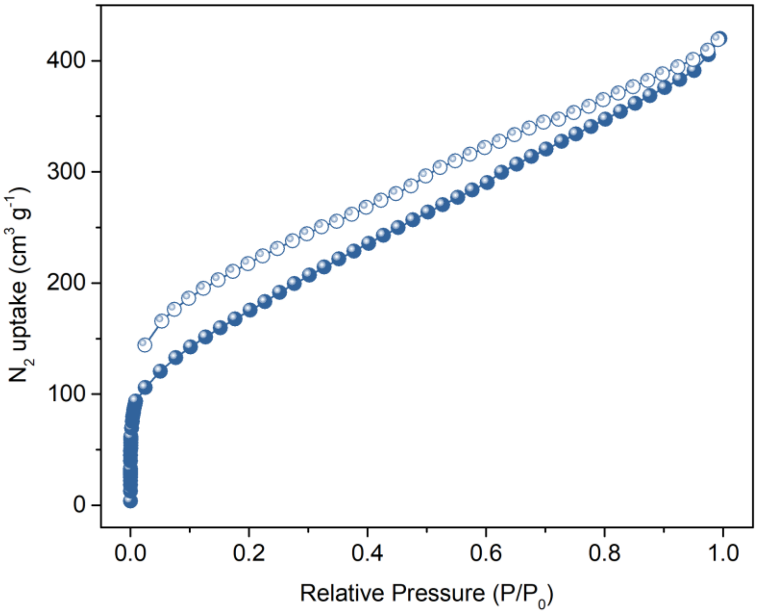

Supplementary Fig. 67. Nitrogen sorption isotherm of COF RICE-5. The presence of hysteresis might be resulted from capillary condensation, which is a common phenomenon in the gas adsorption process.

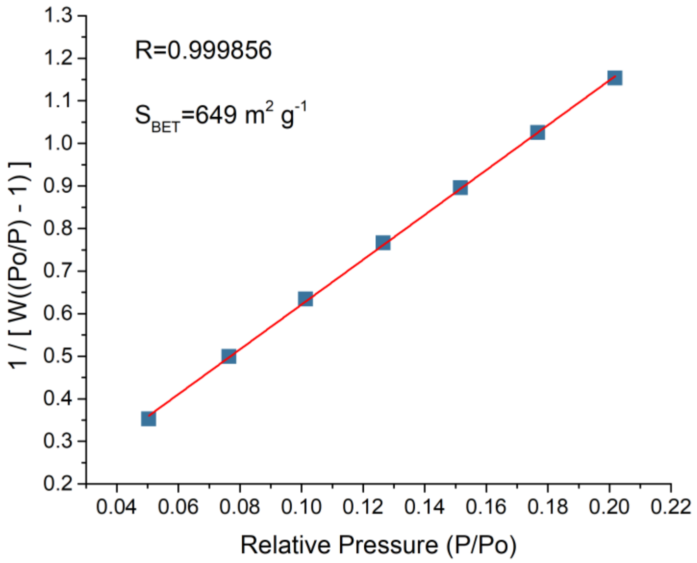

Supplementary Fig. 68. BET linear fitting plot for COF RICE-5.

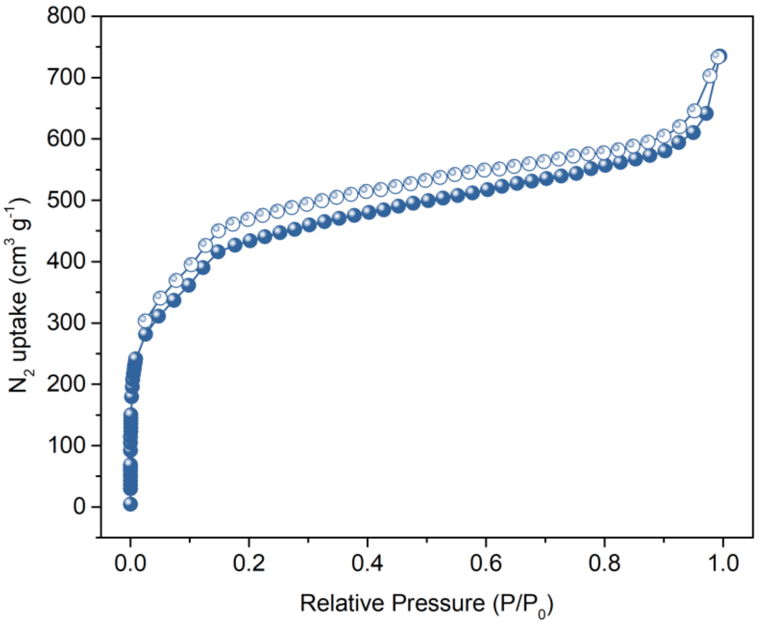

Supplementary Fig. 69. Nitrogen sorption isotherm of COF RICE-6.

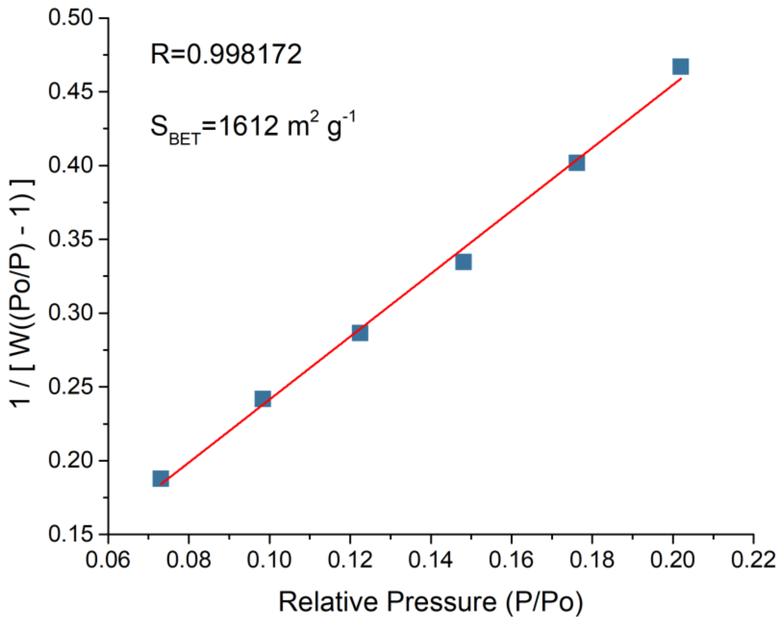

Supplementary Fig. 70. BET linear fitting plot for COF RICE-6.

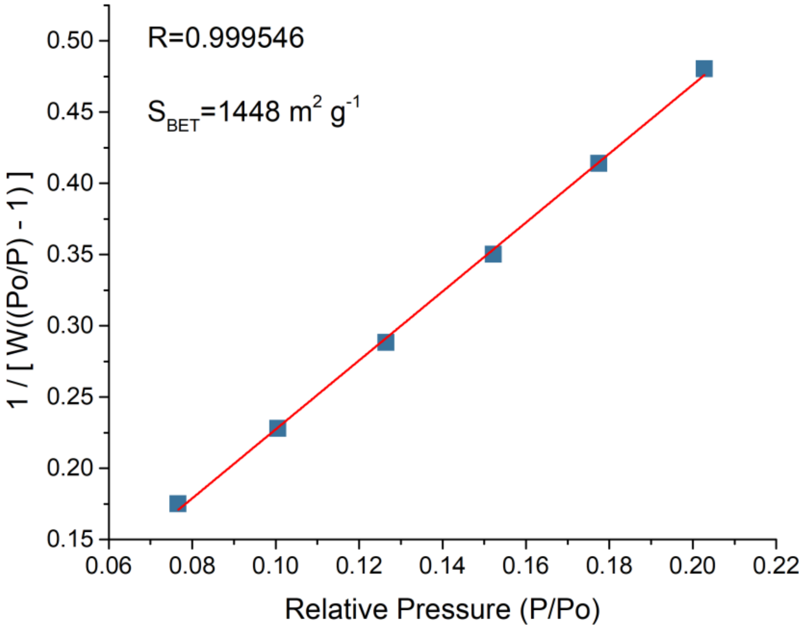

Supplementary Fig. 71. BET linear fitting plot for COF RICE-7.

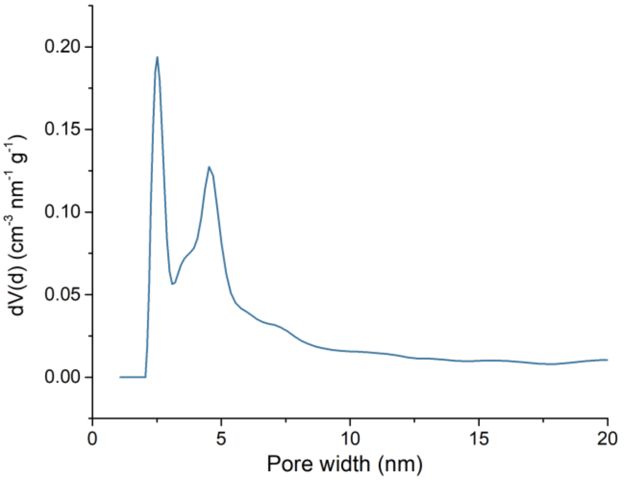

Supplementary Fig. 72. Pore size distributions for COF RICE-4.

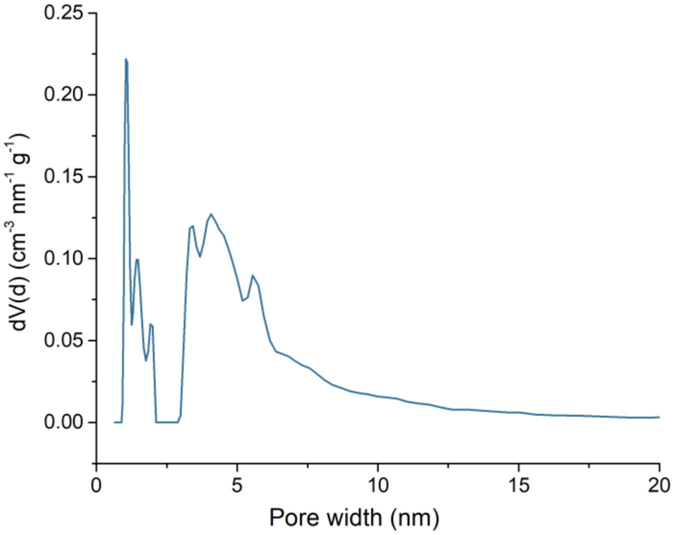

Supplementary Fig. 73. Pore size distributions for COF RICE-5.

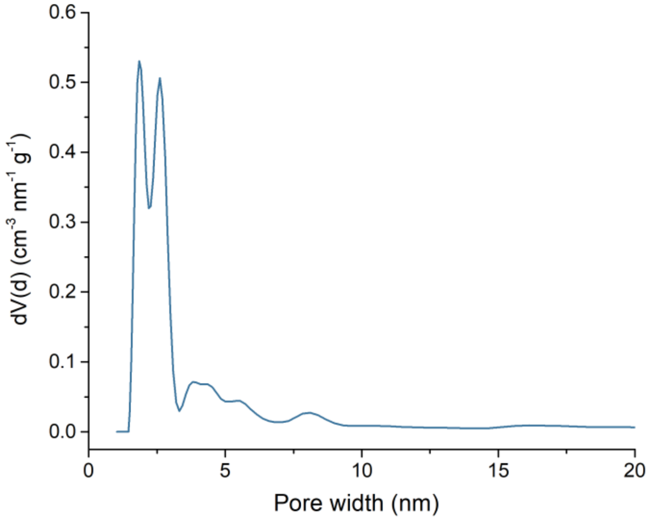

Supplementary Fig. 74. Pore size distributions for COF RICE-6.

^{1}H-NMR of digested COFs

Digestion of COF RICE-3 and ^{1}H NMR test

To probe the monomer ratios in COF-RICE-3, dry COF powders were digested in a glass vial using a solvent mixture of DMSO-*d*6 and deuterium chloride solution (35 wt. % in D_2O, ≥99 atom % D) (v/v, 10/1). To note, we tried multiple solvent conditions to digest the COF powders. RICE-3 could not be digested using solvent mixtures including trifluoroacetic acid/water and formic acid/water even at 80 °C. RICE-3 could be digested using DMSO and NaOH at 60 °C and could not be digested at room temperature. However, NaOH precipitated out at room temperature, and it was not convenient, so we chose the solvent mixture of DMSO and hydrochloric acid as the digestion solvent. The vial was sonicated and then heated on a hot plate at 80 °C until the solution was clear. Afterward, the clear solution was transferred into an NMR tube and tested for ^{1}H NMR. The result was shown in Supplementary Fig. 75, and the peaks were indexed. We used signal e and signal 3 to calculate the ratio of aldehyde and amine monomers. Signal e has 4 units of intensity, while signal 3 has 8.24 units of intensity from two protons in the amine. The aldehyde has 4 equivalent aldehyde protons, and the amine has 6 equivalent protons in the 3-position. So, the ratio of aldehyde monomer to amine monomer is ((4/4): (8.24/6)) = 1:1.37, which is approximately 3:4.

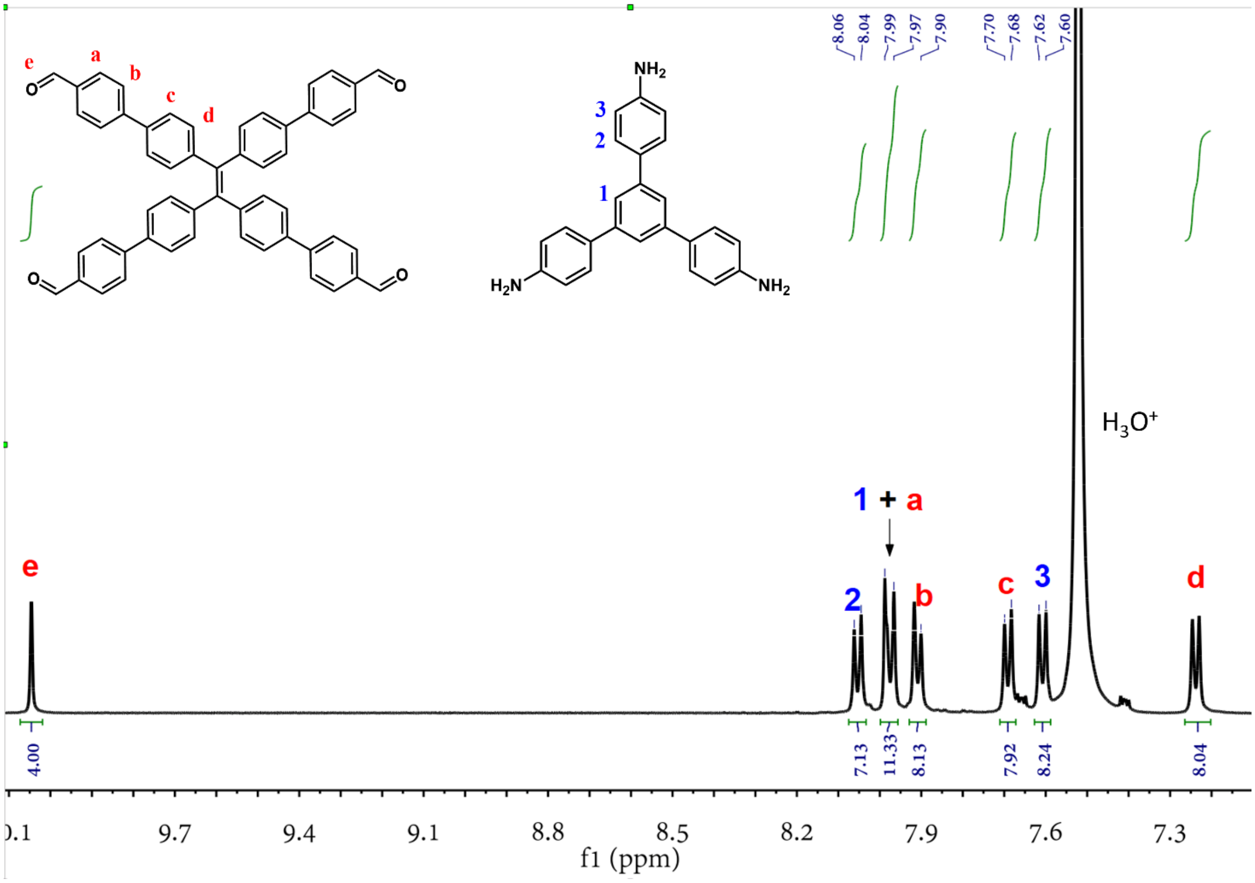

Supplementary Fig. 75. ^{1}H NMR spectrum of digested COF RICE-3 samples

Digestion of COF RICE-7 and ^{1}H NMR test

To probe the monomer ratios in COF-RICE-7, dry COF powders were digested in a glass vial using 1 mL DMSO-d6, 0.1 mL deuterium oxide (99.9 atom % D), and 40 mg NaOH solids. The vial was sonicated and then heated on a hot plate at 80 °C until the solution was clear. Afterward, the clear solution was transferred into an NMR tube and tested for ^{1}H NMR. To prevent the NaOH solids precipitate out, the NMR tube was kept warm using a hot cotton cloth before the test. To note, we also tried multiple solvent mixtures to digest RICE-7. The digestion of RICE-7 was more challenging than that of RICE-3. RICE-7 could not be digested using formic acid/water even at 80 °C, where the solution was cloudy and suspended sheets existed. In the solvent mixtures of trifluoroacetic acid and water with water contents ranging from 5 v% to 20 v%, RICE-7 could not be digested even at 80 °C, and many particles existed. Similarly, RICE-7 could not be digested using DMSO/35 wt% HCl both at room temperature and 80 °C, and particles existed in the solution. The combination of DMSO and NaOH was the only solvent found that could digest RICE-7 at 80 °C.

The result was shown in Supplementary Fig. 76 and the peaks were indexed (unassigned peaks may come from oligomers and Cannizzaro reaction might have occurred in the base conditions). We use peak "a" as a standard (assume we have 1 mol amine), so the integration of a is 8 mol, the theoretical integration of c is 2 mol, and the integration of 1+2+3 is 11.28+10.83-2=20.11 mol. The ratio of amine and aldehyde is 1: (20.11/(6+6+3))= 1:1.34, very close to 3:4.

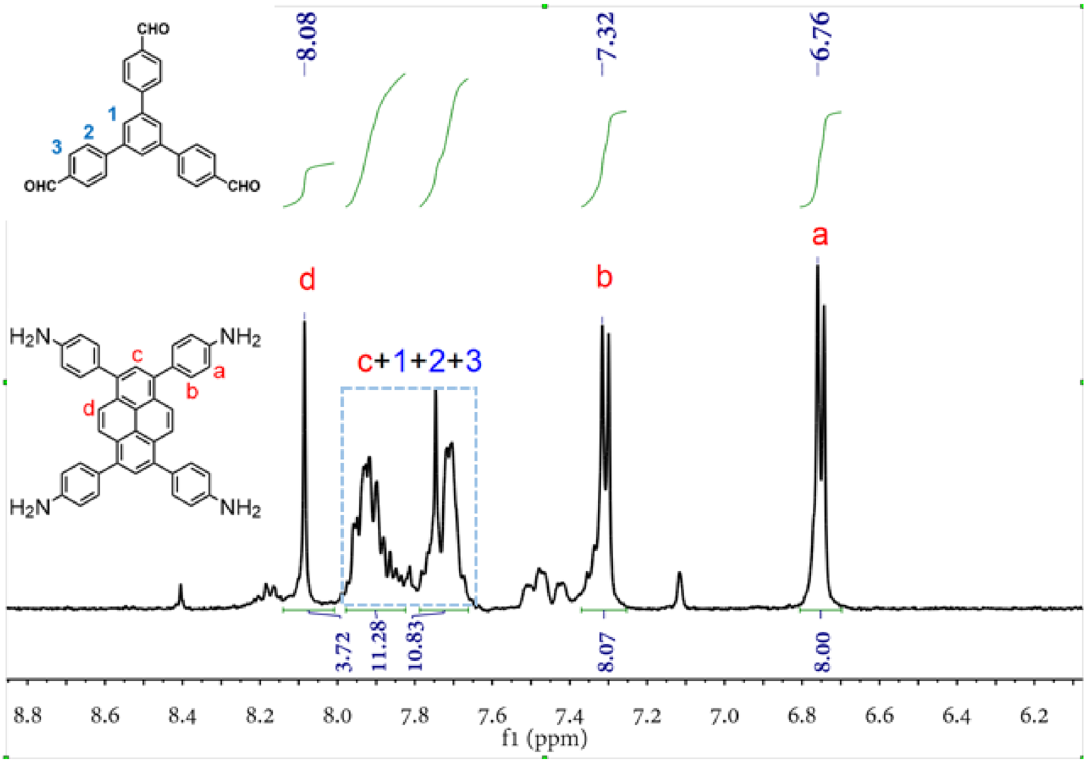

Supplementary Fig. 76. ^{1}H NMR spectrum of digested RICE-7 samples

Stability analysis for typical examples

We studied the chemical stability of RICE-COFs by immersing the COF powders in different solvents for 3 days. After immersion, the COFs were washed, dried, and tested for PXRDs (Supplementary Figs. 77 – 79) and FTIRs (Supplementary Figs. 80 – 82). All the COFs studied showcased high chemical stability as indicated by the consistent PXRD and FTIR measurements.

We also analyzed the pore collapse stability of RICE-3 and RICE-4 to see if the methyl groups on the side functionalities have significant influence on their pore collapse stability. Supplementary Fig. 83 indicates that RICE-3 and RICE-4 are stable upon activation with hexane and ethanol, which have lower surface tension solvents, and they collapse after activation with THF which has high surface tension. This result indicates that RICE-3/4 showcase similar pore collapse stability possibly because the small size of the methyl group does not have a significant impact on the structural rigidity of COFs.

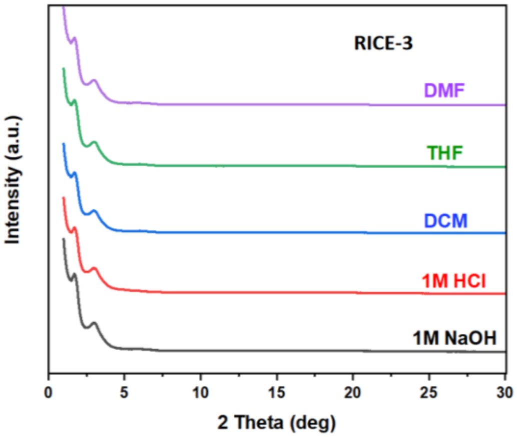

Supplementary Fig. 77. PXRD patterns for COF RICE-3 after immersion in different solvents for 3 days.

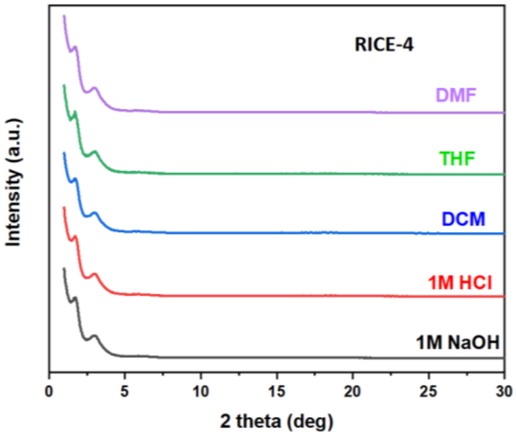

Supplementary Fig. 78. PXRD patterns for COF RICE-4 after immersion in different solvents for 3 days.

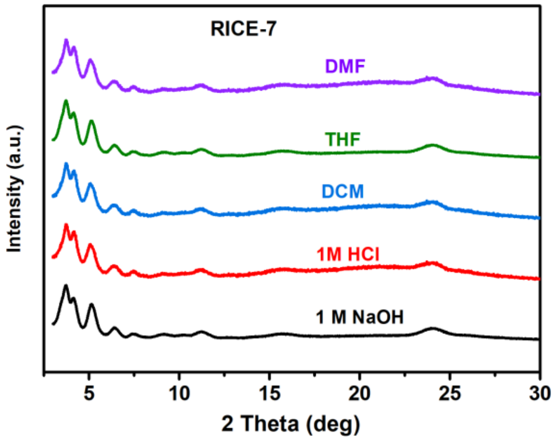

Supplementary Fig. 79. PXRD patterns for COF RICE-7 after immersion in different solvents for 3 days.

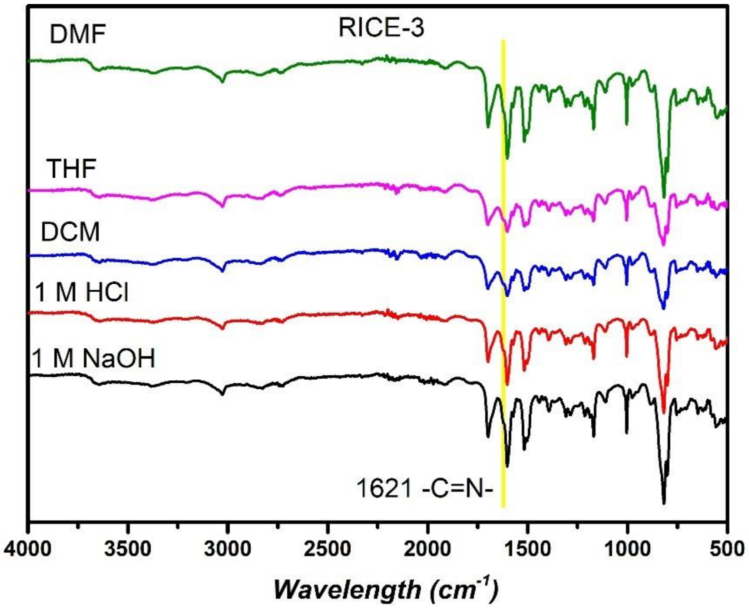

Supplementary Fig. 80. FTIR for COF RICE-3 after immersion in different solvents for 3 days.

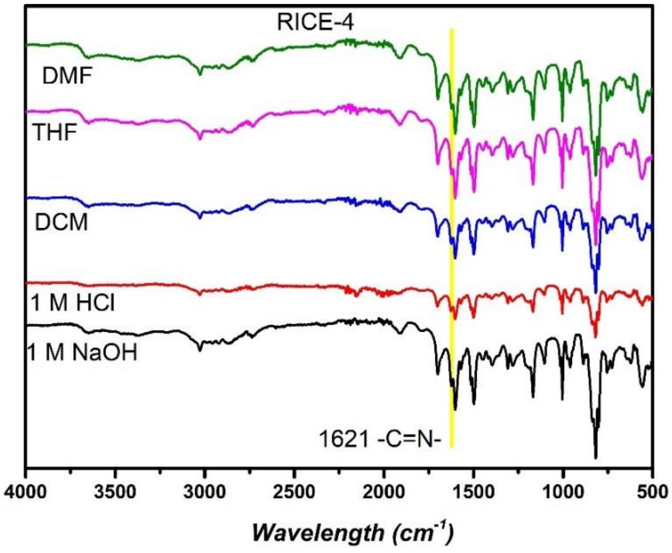

Supplementary Fig. 81. FTIR for COF RICE-4 after immersion in different solvents for 3 days.

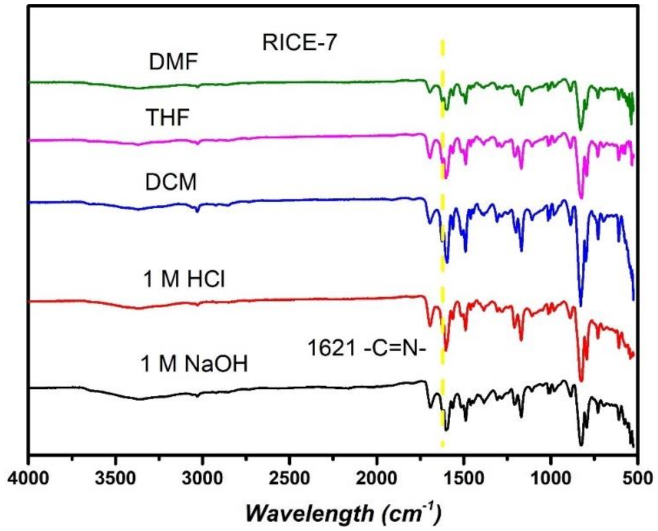

Supplementary Fig. 82. FTIR for COF RICE-7 after immersion in different solvents for 3 days.

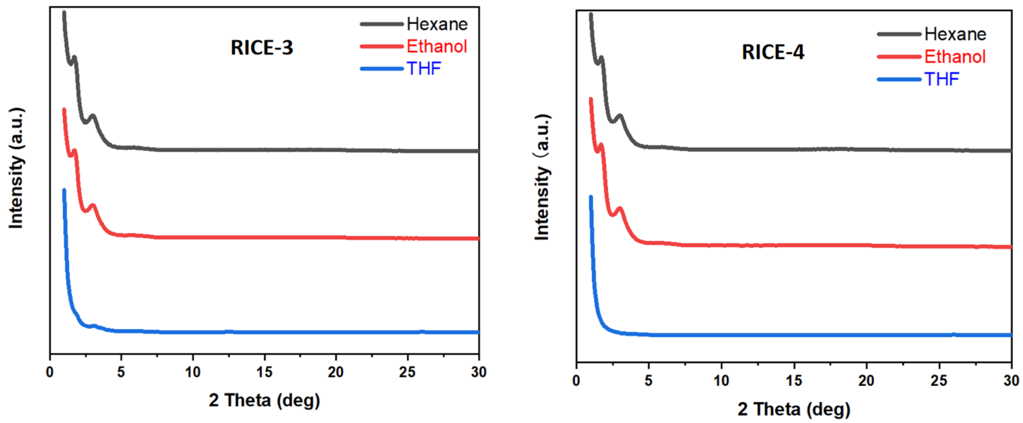

Supplementary Fig. 83. PXRD patterns for the solvent exposure tests of RICE-3/4.

Supplementary Tab. 1. Mesoporous pore sizes in reported 3D COFs.

Compound	BET (m^2 g^{-1})	Largest pore size (nm)	Topology	Ref.
RICE-3	720	4.6	**pto**	This work
RICE-4	654	4.5	**pto**	
RICE-5	649	4.6	**pto**	
RICE-6	1612	4.4	**pto**	
3D-bor-COF-1	3205.5	3.1	**bor**	11
3D-bor-COF-2	1752.7	3.6	**bor**	
3D-bor-COF-3	2077.3	3.8	**bor**	
JUC-551	1728	2.2	**dia**	
JUC-552	3023	2.65	**dia**	
3D pts COF	3478	2.1	**pts**	12
Trip-COF-1	1473	4.0	**stp**	13
Trip-COF-2	1624	2.93	**stp**	
JUC-560	1815	2.5	**ffc**	14
JUC-561	2359	2.46	**ffc**	
COF-790	2650	2.0	**fjh**	15
COF-791	1920	2.28	**fjh**	
COF-792	2250	2.24	**fjh**	

Supplementary Tab. 2. Low-density records in reported MOF/COF materials.

Compound	BET (m^2 g^{-1})	Density (g/cm^3)	Ref.
**RICE-3**	**720**	**0.0948**	**This work**
**RICE-4**	**654**	**0.0943**	
**RICE-5**	**649**	**0.0944**	
**RICE-6**	**1612**	**0.0995**	
COF: JUC-564	3300	0.108	[16]
COF: JUC-518	3018	0.19	[17]
COF-108	n.d	0.17	[18]
DBA-3D-COF-1	5083	0.13	[19]
IRMOF-74-XI	1920	0.195	[20]
MOF-200	4530	0.22	[21]
NU-1301	4748	0.124	[22]

CO_2 adsorption analysis

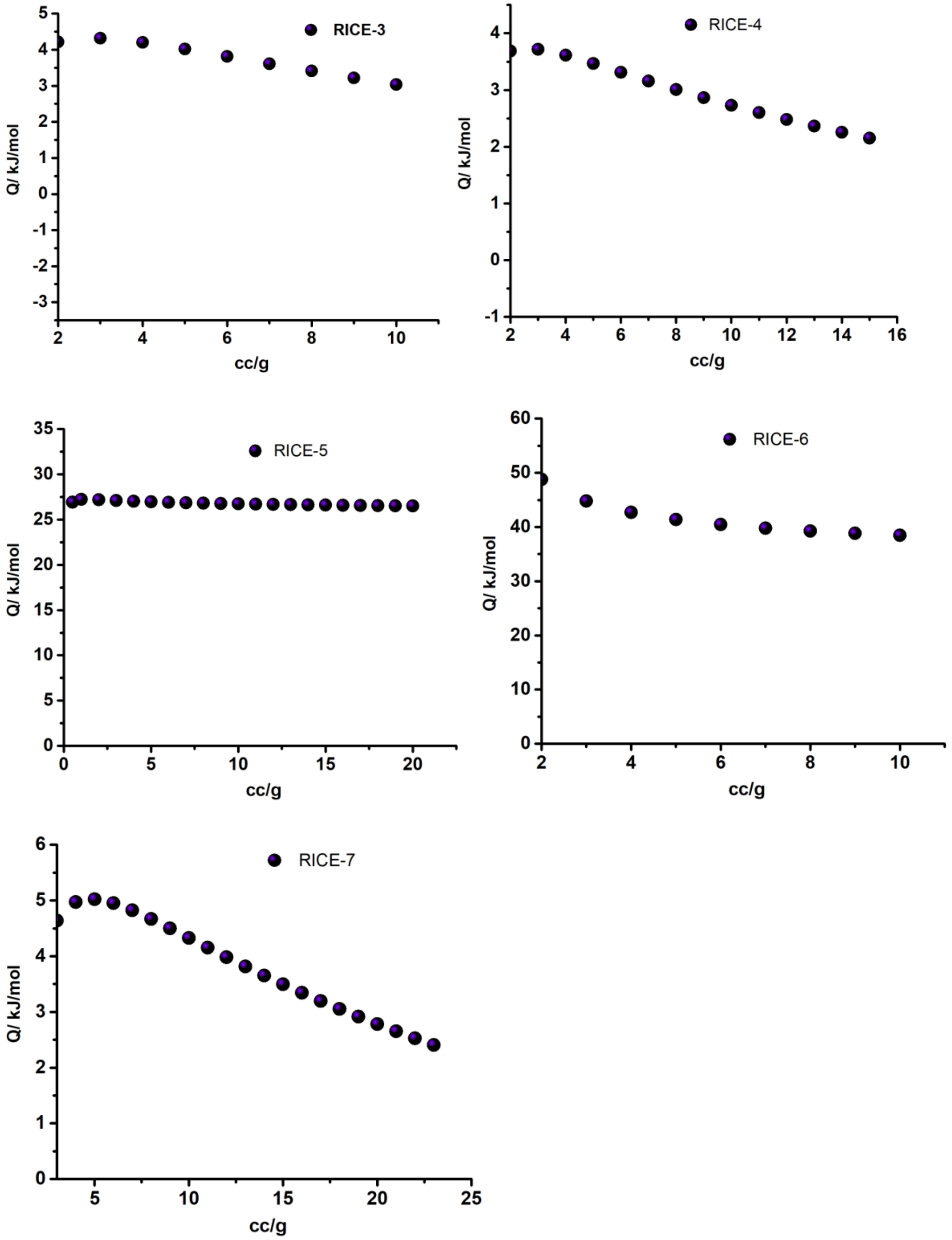

Supplementary Fig. 84. Heat of CO_2 adsorption for RICE-COFs.

DFT calculation of CO_2 adsorption. We studied CO_2 adsorption in the COF RICE-7 structure using density functional theory (DFT). Several candidate adsorption sites were evaluated including both chemisorption and physisorption mechanisms. Within our trials, the chemical bonds between the chemisorbed CO_2 and RICE-7 always broke as the geometry optimization progressed, resulting in the formation of the linear CO_2 molecule. This indicates that CO_2 chemisorption via the formation of chemical bonds with the COF framework is not feasible. Thus, CO_2 adsorption in COF occurs through physisorption mediated by van der Waals interactions. We set the energy of the CO_2 molecule positioned at the center of the largest hole in RICE-7 as the reference state for computed adsorption energies, as the CO_2 molecule in the position does not interact with the COF framework (i.e., because the two are separated by at least13 Å) (**Supplementary Fig. 85a**). When CO_2 is put in a smaller pore in closer proximity to the conjugated COF structure, then the computed adsorption energy is -0.12 eV, as shown in **Supplementary Fig. 85b**. Thus, CO_2 can be stabilized by van der Waals interactions within the COF material. Moreover, when the orientation of the C=O bond of the CO_2 molecule is parallel to the C=C bonds and C=N bonds in the COF structure (**Supplementary Fig. 85c**), then the adsorption is even more favorable (-0.45 eV). This is attributed to the interaction between the CO_2 molecule and the electron-rich, conjugated p orbitals of the COF framework. This adsorption strength is comparable to CO_2 adsorption in zeolites[23] and Metal–Organic Frameworks (MOFs)[24,25], which are known CO_2 absorbents. These calculations show that RICE-7 is a suitable material for CO_2 capture applications.

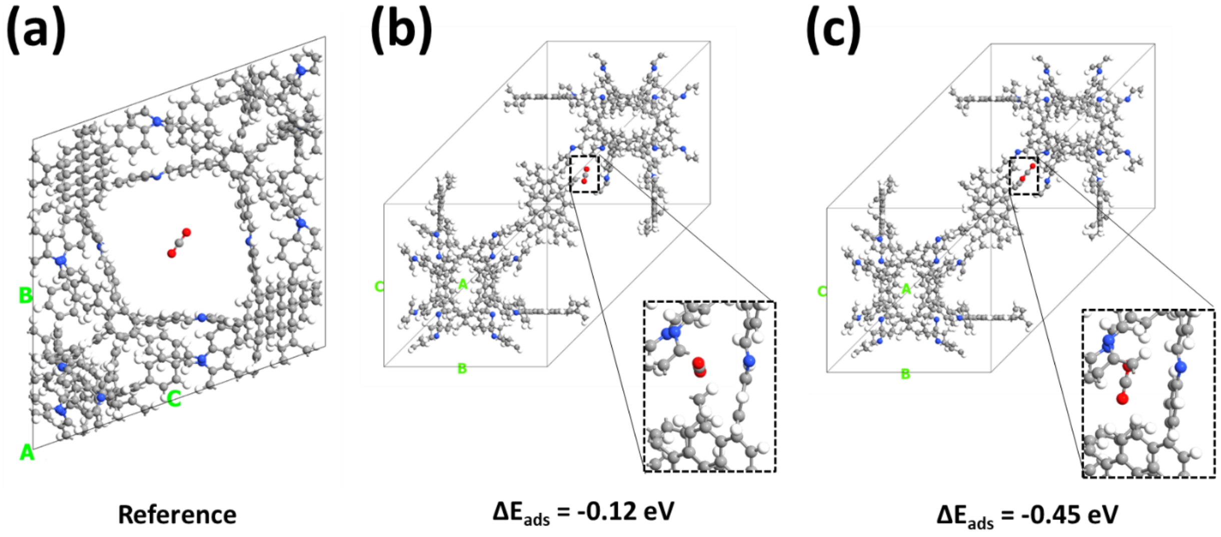

Supplementary Fig. 85. Representative CO_2 adsorption configurations in the COF RICE-7 structure at three different pore sites. O, C, N, and H are shown as red, grey, blue, and white spheres, respectively. The adsorption energies were calculated for three different positions (a, b, and c) and referenced to the position shown in a.

Fractional atomic coordinates and unit cell parameters

Supplementary Tab. 3. Fractional atomic coordinates for simulated RICE-3

Space group: PM-3 (NO.200)			
3D cubic; $a = b = c = 49.420$ Å; $\alpha = \beta = \gamma = 90°$			
Atom	x	y	z
C1	-0.02892	0.5256	-0.14338
C2	-0.03395	0.53699	-0.16885
C3	-0.04467	0.56303	-0.1709
C4	-0.05074	0.57795	-0.14749
C5	-0.04665	0.56608	-0.12197
C6	-0.03562	0.54007	-0.11994
C7	-0.06049	0.60615	-0.14985
C8	-0.08318	0.61494	-0.1352
C9	-0.09288	0.64136	-0.13839
C10	-0.07985	0.65944	-0.15607
C11	-0.05676	0.65083	-0.17014
C12	-0.04722	0.62438	-0.16715
C13	-0.09042	0.68697	-0.16074
N14	-0.11386	0.6942	-0.15125
C15	-0.15381	0.76886	-0.16693
C16	-0.12541	0.76759	-0.16797

C17	-0.11189	0.74334	-0.16237
C18	-0.12662	0.71991	-0.15573
C19	-0.15477	0.72144	-0.15365
C20	-0.16835	0.7456	-0.15936
C21	0.82537	0.8317	0.79401
C22	0.8399	0.80882	0.80254
H23	-0.02858	0.52608	-0.18707
H24	-0.04798	0.5717	-0.19075
H25	-0.051	0.57735	-0.1037
H26	-0.03145	0.53155	-0.10019
H27	-0.09375	0.60115	-0.12184
H28	-0.11072	0.64766	-0.12732
H29	-0.04634	0.66446	-0.18378
H30	-0.02941	0.61811	-0.17828
H31	-0.07904	0.70035	-0.17383
H32	-0.11369	0.7854	-0.17306
H33	-0.09005	0.74306	-0.16303
H34	-0.16616	0.7035	-0.14836
H35	-0.19025	0.74586	-0.15869
H36	0.85732	0.8022	0.79104
C37	-0.01372	0.5	-0.14146

Supplementary Tab. 4. Fractional atomic coordinates for simulated RICE-7

Space group: F23 (NO.196)			
3D cubic; $a = b = c = 46.01$ Å; $\alpha = \beta = \gamma = 90°$			
Atom	x	y	z
C1	0.03713	0.51785	0.34272
C2	0.02845	0.54718	0.34358
C3	0.06717	0.5098	0.34211
C4	0.09195	0.5315	0.34126
C5	0.0947	0.5518	0.31849
C6	0.11893	0.57062	0.31676
C7	0.14141	0.56953	0.33773
C8	0.13856	0.54924	0.36038
C9	0.11434	0.53048	0.36214
N10	0.16768	0.58734	0.33635
C11	0.17316	0.60842	0.31798
C12	0.25419	0.6577	0.31253
C13	0.24993	0.63779	0.33549
C14	0.22389	0.62142	0.33765
C15	0.20141	0.62496	0.31712

C16	0.20585	0.64507	0.29453
C17	0.23178	0.66102	0.29211
C18	-0.0151	0.50404	0.34288
C19	-0.0231	0.53397	0.34283
C20	-0.05301	0.54205	0.342
C21	-9.2E-4	0.55505	0.34325
C22	-0.06309	0.57314	0.33975
C23	-0.07602	0.58317	0.31397
C24	-0.08708	0.61163	0.31181
C25	-0.0862	0.63087	0.33577
C26	-0.07284	0.62089	0.36137
C27	-0.0614	0.59244	0.36345
N28	-0.09846	0.66016	0.33509
C29	-0.11491	0.67081	0.31443
C30	-0.15633	0.75599	0.31285
C31	-0.13604	0.74946	0.33492
C32	-0.12177	0.72213	0.33565
C33	-0.12818	0.70063	0.3148
C34	-0.14833	0.70757	0.29293
C35	-0.16204	0.73472	0.2918
C36	-0.0743	0.52002	0.3417
C37	0.28629	0.69946	0.32868

C38	0.2813	0.67574	0.30982
C39	-0.17304	0.78394	0.3119
C40	-0.16895	0.80344	0.28857
H41	0.04439	0.56443	0.34458
H42	0.07827	0.55267	0.30164
H43	0.12005	0.58541	0.29862
H44	0.15552	0.54781	0.37661
H45	0.11314	0.51493	0.37979
H46	0.15708	0.61447	0.30198
H47	0.26684	0.63485	0.3516
H48	0.22119	0.60608	0.3553
H49	0.18904	0.64836	0.27854
H50	0.23422	0.67639	0.27444
H51	-0.00617	0.57799	0.34333
H52	-0.07751	0.5688	0.29536
H53	-0.09603	0.61824	0.29122
H54	-0.07177	0.63527	0.37988
H55	-0.05177	0.58527	0.38371
H56	-0.12065	0.65745	0.29609
H57	-0.13126	0.76551	0.35146
H58	-0.10599	0.71757	0.35249
H59	-0.15378	0.69157	0.27675

H60	-0.17785	0.73888	0.27486
H61	-0.097	0.52626	0.34065
H62	0.27131	0.70362	0.34632
H63	-0.15175	0.79922	0.27311

REFERENCES

(1) Kresse, G.; Furthmüller, J. Efficient Iterative Schemes for Ab Initio Total-Energy Calculations Using a Plane-Wave Basis Set. *Phys. Rev. B* **1996**, *54* (16), 11169–11186. https://doi.org/10.1103/PhysRevB.54.11169.

(2) Kresse, G.; Furthmüller, J. Efficiency of Ab-Initio Total Energy Calculations for Metals and Semiconductors Using a Plane-Wave Basis Set. *Comput. Mater. Sci.* **1996**, *6* (1), 15–50. https://doi.org/10.1016/0927-0256(96)00008-0.

(3) Perdew, J. P.; Burke, K.; Ernzerhof, M. Generalized Gradient Approximation Made Simple. *Phys. Rev. Lett.* **1996**, *77* (18), 3865–3868. https://doi.org/10.1103/PhysRevLett.77.3865.

(4) Kresse, G.; Joubert, D. From Ultrasoft Pseudopotentials to the Projector Augmented-Wave Method. *Phys. Rev. B* **1999**, *59* (3), 1758–1775. https://doi.org/10.1103/PhysRevB.59.1758.

(5) Blöchl, P. E. Projector Augmented-Wave Method. *Phys. Rev. B* **1994**, *50* (24), 17953–17979. https://doi.org/10.1103/PhysRevB.50.17953.

(6) Grimme, S.; Antony, J.; Ehrlich, S.; Krieg, H. A Consistent and Accurate Ab Initio Parametrization of Density Functional Dispersion Correction (DFT-D) for the 94 Elements H-Pu. *J. Chem. Phys.* **2010**, *132* (15), 154104. https://doi.org/10.1063/1.3382344.

(7) Grimme, S. Semiempirical GGA-type density functional constructed with a long-range dispersion correction. *J. Comput. Chem.* **2006**, *27* (15), 1787–1799. https://doi.org/10.1002/jcc.20495.

(8) Bučko, T.; Hafner, J.; Lebègue, S.; Ángyán, J. G. Improved Description of the Structure of Molecular and Layered Crystals: Ab Initio DFT Calculations with van Der Waals Corrections. *J. Phys. Chem. A* **2010**, *114* (43), 11814–11824. https://doi.org/10.1021/jp106469x.

(9) Grimme, S.; Ehrlich, S.; Goerigk, L. Effect of the Damping Function in Dispersion Corrected Density Functional Theory. *J. Comput. Chem.* **2011**, *32* (7), 1456–1465. https://doi.org/10.1002/jcc.21759.

(10) Monkhorst, H. J.; Pack, J. D. Special Points for Brillouin-Zone Integrations. *Phys. Rev. B* **1976**, *13* (12), 5188–5192. https://doi.org/10.1103/PhysRevB.13.5188.

(11) Li, Z.; Hsueh, C.; Tang, Z.; Chen, J.; Wang, X.; Cui, H.; Yang, Y.; Wang, X.; Ren, D.; Gao, H.; Li, M.; Xu, H.; He, X. Rational Design of Imine-Linked Three-Dimensional Mesoporous Covalent Organic Frameworks with Bor Topology. *SusMat* **2022**, *2* (2), 197–205. https://doi.org/10.1002/sus2.54.

(12) Chen, L.; Gong, C.; Wang, X.; Dai, F.; Huang, M.; Wu, X.; Lu, C.-Z.; Peng, Y. Substoichiometric 3D Covalent Organic Frameworks Based on Hexagonal Linkers. *J. Am. Chem. Soc.* **2021**, *143* (27), 10243–10249. https://doi.org/10.1021/jacs.1c03739.

(13) Wang, Y.; Wu, C.; Sun, W.; Pan, Q.; Hao, W.; Liu, H.; Sun, J.; Li, Z.; Sun, J.; Zhao, Y. Triptycene-Based Three-Dimensional Covalent Organic Frameworks with **Stp** Topology of

Honeycomb Structure. *Mater. Chem. Front.* **2021**, *5* (2), 944–949. https://doi.org/10.1039/D0QM00846J.
(14) Chang, J.; Li, H.; Zhao, J.; Guan, X.; Li, C.; Yu, G.; Valtchev, V.; Yan, Y.; Qiu, S.; Fang, Q. Tetrathiafulvalene-Based Covalent Organic Frameworks for Ultrahigh Iodine Capture. *Chem. Sci.* **2021**, *12* (24), 8452–8457. https://doi.org/10.1039/D1SC01742J.
(15) Nguyen, H. L.; Gropp, C.; Ma, Y.; Zhu, C.; Yaghi, O. M. 3D Covalent Organic Frameworks Selectively Crystallized through Conformational Design. *J. Am. Chem. Soc.* **2020**, *142* (48), 20335–20339. https://doi.org/10.1021/jacs.0c11064.
(16) Li, H.; Ding, J.; Guan, X.; Chen, F.; Li, C.; Zhu, L.; Xue, M.; Yuan, D.; Valtchev, V.; Yan, Y.; Qiu, S.; Fang, Q. Three-Dimensional Large-Pore Covalent Organic Framework with Stp Topology. *J. Am. Chem. Soc.* **2020**, *142* (31), 13334–13338. https://doi.org/10.1021/jacs.0c06485.
(17) Li, H.; Chang, J.; Li, S.; Guan, X.; Li, D.; Li, C.; Tang, L.; Xue, M.; Yan, Y.; Valtchev, V.; Qiu, S.; Fang, Q. Three-Dimensional Tetrathiafulvalene-Based Covalent Organic Frameworks for Tunable Electrical Conductivity. *J. Am. Chem. Soc.* **2019**, *141* (34), 13324–13329. https://doi.org/10.1021/jacs.9b06908.
(18) El-Kaderi, H. M.; Hunt, J. R.; Mendoza-Cortés, J. L.; Côté, A. P.; Taylor, R. E.; O'Keeffe, M.; Yaghi, O. M. Designed Synthesis of 3D Covalent Organic Frameworks. *Science* **2007**, *316* (5822), 268–272. https://doi.org/10.1126/science.1139915.
(19) Baldwin, L. A.; Crowe, J. W.; Pyles, D. A.; McGrier, P. L. Metalation of a Mesoporous Three-Dimensional Covalent Organic Framework. *J. Am. Chem. Soc.* **2016**, *138* (46), 15134–15137. https://doi.org/10.1021/jacs.6b10316.
(20) Deng, H.; Grunder, S.; Cordova, K. E.; Valente, C.; Furukawa, H.; Hmadeh, M.; Gándara, F.; Whalley, A. C.; Liu, Z.; Asahina, S.; Kazumori, H.; O'Keeffe, M.; Terasaki, O.; Stoddart, J. F.; Yaghi, O. M. Large-Pore Apertures in a Series of Metal-Organic Frameworks. *Science* **2012**, *336* (6084), 1018–1023. https://doi.org/10.1126/science.1220131.
(21) Furukawa, H.; Ko, N.; Go, Y. B.; Aratani, N.; Choi, S. B.; Choi, E.; Yazaydin, A. Ö.; Snurr, R. Q.; O'Keeffe, M.; Kim, J.; Yaghi, O. M. Ultrahigh Porosity in Metal-Organic Frameworks. *Science* **2010**, *329* (5990), 424–428. https://doi.org/10.1126/science.1192160.
(22) Li, P.; Vermeulen, N. A.; Malliakas, C. D.; Gómez-Gualdrón, D. A.; Howarth, A. J.; Mehdi, B. L.; Dohnalkova, A.; Browning, N. D.; O'Keeffe, M.; Farha, O. K. Bottom-up Construction of a Superstructure in a Porous Uranium-Organic Crystal. *Science* **2017**, *356* (6338), 624–627. https://doi.org/10.1126/science.aam7851.
(23) Fang, H.; Kamakoti, P.; Zang, J.; Cundy, S.; Paur, C.; Ravikovitch, P. I.; Sholl, D. S. Prediction of CO2 Adsorption Properties in Zeolites Using Force Fields Derived from Periodic Dispersion-Corrected DFT Calculations. *J. Phys. Chem. C* **2012**, *116* (19), 10692–10701. https://doi.org/10.1021/jp302433b.
(24) Poloni, R.; Lee, K.; Berger, R. F.; Smit, B.; Neaton, J. B. Understanding Trends in CO2 Adsorption in Metal–Organic Frameworks with Open-Metal Sites. *J. Phys. Chem. Lett.* **2014**, *5* (5), 861–865. https://doi.org/10.1021/jz500202x.
(25) Queen, W. L.; Hudson, M. R.; Bloch, E. D.; Mason, J. A.; Gonzalez, M. I.; Lee, J. S.; Gygi, D.; Howe, J. D.; Lee, K.; Darwish, T. A.; James, M.; Peterson, V. K.; Teat, S. J.; Smit, B.; Neaton, J. B.; Long, J. R.; Brown, C. M. Comprehensive Study of Carbon Dioxide Adsorption in the Metal–Organic Frameworks M2(Dobdc) (M = Mg, Mn, Fe, Co, Ni, Cu, Zn). *Chem. Sci.* **2014**, *5* (12), 4569–4581. https://doi.org/10.1039/C4SC02064B.
